# Probing substrate water access through the O1 channel of Photosystem II by single site mutations and membrane inlet mass spectrometry

**DOI:** 10.1007/s11120-025-01147-4

**Published:** 2025-04-22

**Authors:** A. Orkun Aydin, Casper de Lichtenberg, Feiyan Liang, Jack Forsman, André T. Graça, Petko Chernev, Shaochun Zhu, André Mateus, Ann Magnuson, Mun Hon Cheah, Wolfgang P. Schröder, Felix Ho, Peter Lindblad, Richard J. Debus, Fikret Mamedov, Johannes Messinger

**Affiliations:** 1 Molecular Biomimetics, Department of Chemistry– Ångström, Uppsala University, Uppsala, 751 20 Sweden; 2 Present Address: Department of Plant and Environmental Sciences, University of Copenhagen, Frederiksberg C, 1871 Denmark; 3 Department of Chemistry, Chemical Biology Centre, Umeå University, Umeå, 907 36 Sweden; 4 Department of Plant Physiology, Umeå Plant Science Center (UPSC), Umeå University, Umeå, 901 87 Sweden; 5 Present Address: European Molecular Biology Laboratory, EMBL Grenoble, Grenoble, 38042 France; 6 The Laboratory for Molecular Infection Medicine Sweden (MIMS), Umeå University, Umeå, 907 36 Sweden; 7 Department of Biochemistry, University of California, Riverside, CA 92521 USA

**Keywords:** Photosystem II, Oxygen evolving complex, Water oxidation, Synechocystis sp. PCC 6803, O1 channel, Water delivery, Substrate water exchange, Water wheel, D1-E329, CP43-V410

## Abstract

Light-driven water oxidation by photosystem II sustains life on Earth by providing the electrons and protons for the reduction of CO_2_ to carbohydrates and the molecular oxygen we breathe. The inorganic core of the oxygen evolving complex is made of the earth-abundant elements manganese, calcium and oxygen (Mn_4_CaO_5_ cluster), and is situated in a binding pocket that is connected to the aqueous surrounding via water-filled channels that allow water intake and proton egress. Recent serial crystallography and infrared spectroscopy studies performed with PSII isolated from *Thermosynechococcus vestitus* (*T. vestitus*) support that one of these channels, the O1 channel, facilitates water access to the Mn_4_CaO_5_ cluster during its S_2_→S_3_ and S_3_→S_4_→S_0_ state transitions, while a subsequent CryoEM study concluded that this channel is blocked in the cyanobacterium *Synechocystis sp.* PCC 6803, questioning the role of the O1 channel in water delivery. Employing site-directed mutagenesis we modified the two O1 channel bottleneck residues D1-E329 and CP43-V410 (*T. vestitus* numbering) and probed water access and substrate exchange via time resolved membrane inlet mass spectrometry. Our data demonstrates that water reaches the Mn_4_CaO_5_ cluster via the O1 channel in both wildtype and mutant PSII. In addition, the detailed analysis provides functional insight into the intricate protein-water-cofactor network near the Mn_4_CaO_5_ cluster that includes the pentameric, near planar ‘water wheel’ of the O1 channel.

**Supplementary Information:**

The online version contains supplementary material available at 10.1007/s11120-025-01147-4.

## Introduction

**Fig. 1:**
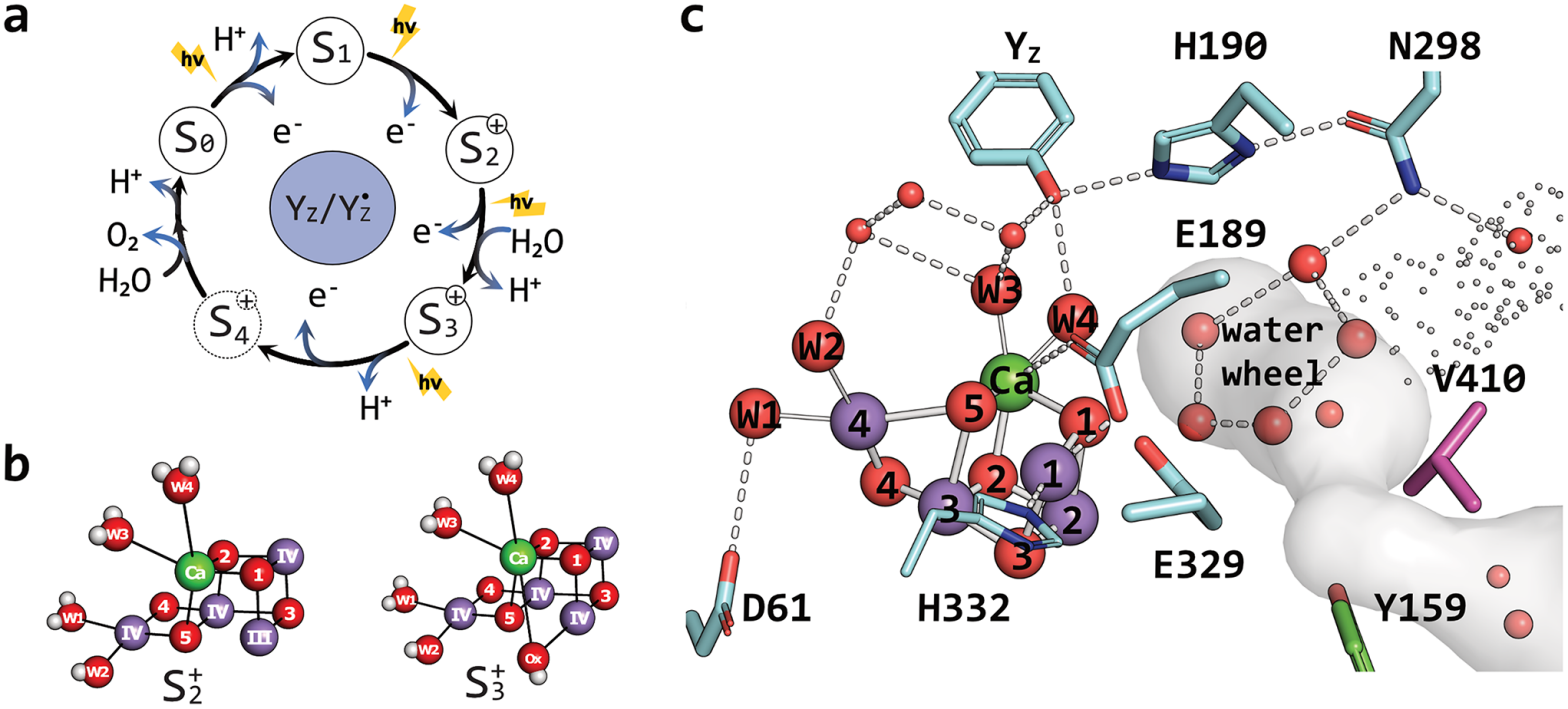
(**a**) Kok-cycle showing substrate/products for each light-induced S-state transition. (**b**) The open cubane structure of the Mn_4_CaO_5_ cluster in S_2_ state and of the Mn_4_CaO_5_-O_X_ structure of the S_3_ state that is reached by water insertion, shown from left to right. (**c**) Active site of the water oxidation in Photosystem II in *Synechocystis* sp. PCC 6803 and its connection to the O1 channel system. Cyan, magenta and green denotes; D1, CP43 and PsbV residues in order. Red spheres show water molecules or oxo-bridges, purple and green spheres show manganese and calcium ions, respectively. Light-green dashed lines are showing potential hydrogen bonds. Grey surface and dots signify the O1 channel network calculated using CAVER 3.0 (Chovancova et al. 2012) on the PDB entry 7N8O. Structure based on PDB 7N8O is prepared using Pymol

Cyanobacteria, algae and higher plants perform the life-sustaining water oxidation reaction in the oxygen-evolving complex (OEC) of Photosystem II (PSII) (Shevela et al. 2023). The inorganic core of the OEC contains four manganese ions (Mn1-Mn4), one calcium ion and five oxo bridges (O1-O5). Mn1, Mn2, Mn3 and Ca, together with four oxo bridges (O1-O3, O5), form an open cuboid that is connected to Mn4 via O5 and O4 (Fig. 1). This Mn_4_CaO_5_ cluster is coordinated by the protein via five carboxylate head groups from amino acid side chains, the C-terminal carboxylate of the D1 protein, and one histidine. Additionally, four water molecules bind to the cluster: W1 and W2 are ligands of Mn4, and W3 and W4 are coordinating the Ca ion (Umena et al. 2011; Kern et al. 2018).

Light absorption by chlorophyll and the transfer of excitation energy into the reaction center results in charge separation, i.e. the formation of an electron hole at the primary donor P680 and the reduction of the primary acceptor pheophytin (Pheo_D1_). Pheo_D1_^●^ subsequently transfers the electron to the two quinones Q_A_ and Q_B_ at the acceptor side of PSII. The highly oxidizing P680^●+^ is reduced by D1-Tyr161 (Y_Z_), and Y_Z_^●^ in turn oxidizes the Mn_4_CaO_5_ cluster. Sequential charge separations cycle the Mn_4_CaO_5_ cluster through four distinct redox intermediates, from S_0_ to S_3_, leading to the evolution of molecular oxygen during the S_3_→S_4_→ S_0_ transition (Kok cycle in Fig. 1a; for an educational review, see Shevela et al. 2023). Through suitable dark-adaptation, all Mn_4_CaO_5_ clusters in the PSII sample can be synchronized into the S_1_ state, allowing S state specific structural and spectroscopic investigations to be performed with well-adjusted flash sequences.

In recent room temperature serial crystallography studies conducted using x-ray free-electron lasers (XFELs), researchers tracked the events during the S_2_→S_3_ transition, unveiling the emergence of a new hydroxo-bridge (O_X/6_; Fig. 1b) between Ca and Mn1 as a consequence of the binding of one water molecule (Suga et al. 2017, 2019; Kern et al. 2018; Ibrahim et al. 2020), validating earlier theoretical and experimental suggestions of the formation of a Mn_4_CaO_5_-O_X_ cluster in the S_3_ state (Siegbahn 2006; Noguchi 2007; Kim and Debus 2019). Excitingly, the XFEL measurements also gave insight into the OEC rearrangements during the S_3_→S_4_→S_0_ transition that facilitate the binding of the second substrate water molecule, the release of two protons and the release of O_2_ (Bhowmick et al. 2023).

The oxidation states of the Mn ions in the S_0_ state of the OEC have been assigned to be Mn_1,2,3,4_(III, IV, III, III) by a combination of EPR and ^55^Mn-ENDOR spectroscopy as well as theoretical studies (Messinger et al. 1997a, b; Kulik et al. 2007; Siegbahn 2009; Krewald et al. 2015). The overall oxidation state assignment, referred to as high oxidation state paradigm, was recently confirmed by a photoactivation study of PSII crystals (Cheah et al. 2020). During each S state transition, one of the Mn(III) ions is oxidized until Mn(IV, IV, IV, IV) is reached in the S_3_ state (Haumann et al. 2005; Dau and Haumann 2008; Cox et al. 2014). High resolution crystal structures indicate the open cubane structure in the S_0_, S_1_ and S_2_ states. However, spectroscopic and computational results indicate the presence of at least one additional conformation of the S_2_ state (Pantazis et al. 2012; Bovi et al. 2013; Shoji et al. 2015; Boussac et al. 2018; Guo et al. 2024). EPR spectra reveal two distinct signals for the S_2_ state at cryogenic temperatures, the low spin (S = 1/2) multiline signal, S_2_^LS^, at g ≈ 2 associated with the open-cubane configuration (Fig. 1b), and a high spin (S = 5/2) signal at g ≈ 4.1, S_2_^HS^, suggested to be indicative of a closed-cubane structure (Pantazis et al. 2012; Isobe et al. 2012; Bovi et al. 2013). However, depending on species and experimental conditions, additional high-spin signals have been observed and the proposed structures of the Mn_4_CaO_5_ cluster that give rise to these high-spin signals remain controversial, with other groups suggesting the protonation of O4 (Corry and O’Malley 2019) or the early binding of O_X_ (Pushkar et al. 2019; de Lichtenberg and Messinger 2020).

Understanding the mechanism of O-O bond formation during the S_3_→S_4_→S_0_ transition requires identification of the two substrate ‘water’ molecules at the Mn_4_CaO_5_(-O_X_) cluster (the term water is used irrespective of the protonation state). Time-resolved membrane inlet mass spectrometry (TR-MIMS) is currently the only technique capable of probing substrate water binding sites in PSII (Messinger et al. 1995; reviewed in Cox and Messinger 2013), and has been more recently complemented by (freeze-quench) ^17^O-EDNMR spectroscopy (Rapatskiy et al. 2012; Navarro et al. 2013; de Lichtenberg et al. 2024). By rapidly increasing H_2_^18^O enrichment in the sample, TR-MIMS can measure the exchange rates of the two bound substrate waters, denoted as W_s_ (slowly exchanging) and W_f_ (fast exchanging), in different S states by following the rise of the ^18^O-label in the O_2_ produced. W_s_ exchange can be monitored in all four stable S-states, while W_f_ exchange is resolvable only in the S_2_ and S_3_ states due to its fast exchange rate (Cox and Messinger 2013).

Combining water exchange data with structural and spectroscopic evidence, O5 has been suggested as the primary candidate for W_s_ (Messinger 2004; Rapatskiy et al. 2012), which has been supported by DFT calculations (Siegbahn 2006, 2009, 2013) and was recently proven by demonstrating comparable exchange rates of W_s_ and O5 in the S_1_ state (de Lichtenberg et al. 2024). The location of the fast-exchanging water molecule remains uncertain in the S_2_ state with W3 and a water W_x_ near the Mn_4_CaO_5_ cluster as prime candidates (Messinger 2004; Noguchi 2007; Siegbahn 2009; Cox and Messinger 2013; Kim and Debus 2019; de Lichtenberg et al. 2021b; Hussein et al. 2021; Bhowmick et al. 2023; Li et al. 2024; Isobe et al. 2024; Chernev et al. 2024). In the S_3_ state, O_X_ is proposed to be W_f_ (for discussion, see de Lichtenberg et al. 2021b).

The activity of the OEC relies on the organization of substrate and product pathways within the protein-water-cofactor matrix. Three water-filled channels, which may facilitate transport of substrate water, protons, or oxygen, have been identified in PSII crystal structures (Ho and Styring 2008; Gabdulkhakov et al. 2009; Frankel et al. 2013; Weisz et al. 2017; Hussein et al. 2023). Steered molecular dynamics (MD) simulations have been used to evaluate the energetics of water permeation, revealing bottleneck regions that pose significant activation barriers to water diffusion in all three channels (Vassiliev et al. 2010, 2012).

The O1 channel is of particular interest due to its terminal point near the Ca-bound water molecules W3 and W4 that seemingly connect the O1 channel to O_X_ in the S_3_ state. This end point of the O1 channel also harbors the structurally conserved pentameric, near planar ‘water wheel’ (Fig. 1c) that appears to be involved in O_X_ binding (Kern et al. 2018; Suga et al. 2019; Ibrahim et al. 2020; Hussein et al. 2021; Bhowmick et al. 2023; Li et al. 2024). The solvent accessibility of the O1 channel has been demonstrated by the presence of molecules such as glycerol and DMSO when added to crystallization buffers (Gabdulkhakov et al. 2009). Furthermore, time-resolved XFEL structures revealed a high mobility of water molecules along the O1 channel, especially in the S_2_→S_3_ transition (Suga et al. 2019; Ibrahim et al. 2020; Hussein et al. 2021; Li et al. 2024). During this transition, the rearrangement of several amino acid residues is detected in parallel to the appearance of O_X_. A recent high-resolution CryoEM structure of light-activated PSII revealed additional, partially occupied water binding sites in the vicinity of Ca and W4, which support water insertion from the O1 channel into the O_X_ position during the S_2_→S_3_ transition (Hussein et al. 2024). While all the above findings support the importance of the O1 channel in facilitating the delivery of substrate water to the OEC, in a recent CryoEM structure of PS II from *Synechocystis sp.* PCC 6803, the B branch of the O1 channel was shown to be narrowed by a tyrosine residue, leading to the proposal that it is blocked in this species (Gisriel et al. 2022), which questions the general water delivery function of the O1 channel.

In this study, we created three site-specific PSII mutants in *Synechocystis sp.* PCC 6803 to modify the constraints of the O1 channel bottleneck (Fig. 1c). We substituted the hydrophilic residue D1-E329 with two hydrophobic residues, D1-E329F and D1-E329L, aiming to narrow the bottleneck by imposing the phenyl ring into the channel cavity, while leucine has a comparable size to glutamate but is uncharged. The CP43-V410 residue was mutated to serine to increase the hydrophilicity of the domain. These mutations were intended to modify the water permeability of the O1 channel without directly affecting the coordination geometry of the OEC. We isolated PSII core complexes from these mutants and employed TR-MIMS to examine substrate exchange kinetics in the S_2_ and S_3_ states. EPR spectroscopy was employed to assess changes in the Mn_4_CaO_5_-cluster homogeneity, while variable fluorescence spectroscopy was used to analyze the effects of O1 channel modifications on charge recombination kinetics.

## Experimental methods

### Production of mutants

For the D1-E329 mutations, the modified genes were expressed using pPMQAK1 (Huang et al. 2010) driven by the promoter psbA2. In detail, PpsbA2 was flanked with EcoRI and XbaI, while the CP47 gene, tagged with 6×His, was flanked with XbaI and PstI. After enzymatic digestions, P*psbA2* and the respective D1 gene were ligated with the pPMQAK1 vector, which was prepared by EcoRI and PstI digestion, using Quick ligase (New England Bio-labs). The resulting vectors were used as template to make D1 mutant-expressing vectors through site-directed mutagenesis. The vectors were transferred individually to WT* *Synechocystis* sp. PCC 6803 (hereafter: *Synechocystis*) cells that lacked all psbA genes and contained His-tagged CP47. This was done using triparental mating as detailed elsewhere (Elhai and Wolk 1988). 50 µg/ml kanamycin was used for selection of the successful transformants, while no kanamycin was added during cultivation.

The CP43-V410 residue in *T. vestitus* corresponds to CP43-V397 in *Synechocystis*; however, we employ the *T. vestitus* numbering for consistency with the literature. The CP43-V410S mutation was constructed as described in (Service et al. 2011). Briefly, the mutation was constructed in an *E. coli* plasmid containing the *C*-terminal 60% of the CP43 coding region plus approximately 350 bp of 3’ downstream DNA plus a 1.2 kb fragment of DNA encoding resistance to kanamycin inserted approximately 170 bp downstream of the 3’ end of the psbC coding region (a larger version of this plasmid was the kind gift of W.F. J. Vermaas of Arizona State University). The mutation-bearing plasmid was transformed with the procedure of Williams (Williams 1988) into a strain of *Synechocystis* that lacks the coding region of the large lumenal loop of CP43 plus approximately 250 bp of 3’ flanking DNA. For all mutants, correct transformants were verified by PCR amplifications.

### Protein purification

Wild type cells of the unicellular cyanobacterium *Synechocystis* and strains expressing modified D1 and CP43 proteins (D1-E329F, D1-E329L and CP43-V410S) were grown in BG11 medium in 5 L glass carboys at 30 °C as described earlier (Liang et al. 2018). Thylakoid membranes were isolated by bead beating (Bead-Beater, BioSpec Product, Bartlesville, OK) and subsequent centrifugation at 50.000xg (de Lichtenberg and Messinger 2020; Kim and Debus 2020). The PSII core complexes were extracted by treatment with 1% (w/v) n-dodecyl β-D-maltoside in the presence of protease inhibitors (1 µM PMSF (Sigma), 1 µM caproic acid (Sigma). 1 µM benzamidine) and 2% agar (w/v) DNAase I (Roche). The protein lysate is loaded on a HisPrep FF 16/10 column (GE Healthcare), washed with 4 column volumes before elution 50 mM L-histidine containing buffer in a single step. The Photosystem II core complexes (PSIIcc) were suspended in 1.2 M betaine, 10% (v/v) glycerol, 50 mM MES-NaOH (pH 6.0), 20 mM CaCl_2_, 5 mM MgCl_2_, 50 mM histidine, 1 mM EDTA, and 0.03% (w/v) n-dodecyl β-D-maltoside. The samples were then concentrated to achieve 1 mg Chl/ml and rapidly frozen in liquid N_2_ in 50–100 mL aliquots and stored at -80^o^C until use. All steps were performed at 4 °C in dim green light. The O_2_ evolution activity was measured with a Clark-type electrode at 25 °C in the presence of 0.2 mM 2,5-dichloro-1,4-benzoquinone (DCBQ) and 2 mM potassium ferricyanide (K_3_[Fe(CN)_6_]). The O_2_ rates were (*n* = 3): 2100 (± 300), 2400 (± 500), 1700 (± 400) and 1000 (± 200) µmol O_2_ / (mg Chl)^−1^ h^− 1^ for WT, E329L. E329F and V410S, respectively. Flash induced O_2_ yields (2 Hz flash frequency) were within ± 20% for all PSIIcc in this study.

### Protein subunit composition of isolated PSIIcc

Protein subunit composition of isolated PSIIcc was determined using a label-free LC-MS/MS approach incorporating a modified SP3 sample-preparation protocol (Hughes et al. 2014, 2019). Briefly, samples were denatured, bound to SpeedBeads (50% ethanol, 2.5% formic acid), digested overnight with trypsin, and desalted for downstream analysis. Peptides were introduced into an Exploris 480 (Thermo) via a Vanquish Neo (Thermo) system equipped with a PEPMAP NEO C18 trapping column (300 μm × 5 mm, 5 μm particle size) and a nanoEaseTM M/Z HSS C18 T3 analytical column (75 μm × 250 mm, 1.8 μm particle size). Data acquisition on Exploris 480 (Thermo Scientific) was carried out using a data dependent method. Survey scans covering the mass range of 375–1500 were acquired at a resolution of 120,000, RF lens of 40% and normalized automatic gain control (AGC) of 300%. Maximum cycling time of 2 s was used to control the number of precursors for tandem-MS/MS (MS2) analysis. Charge states include 2–6 charges. Dynamic exclusion was set to exclude the previously selected precursors for 35 s. MS2 scans were acquired at a resolution of 15,000 (at m/z 200), with AGC target value of auto. The isolation window was 1.4 m/z. HCD fragmentation was induced with a normalized collision energy (NCE) of 30. Isotopes were excluded for MS2 analysis.

Raw data was searched against Synechocystis sp. (strain PCC 6803 / Kazusa) UniProt FASTA (proteome identifier [ID] UP000001425) using FragPipe (version 18), label free quantification was achieved using LFQ-MBR workflow. Proteins identified from contaminants and decoys were removed. MaxLFQ Intensity of the three most abundant peptides per protein was used to estimate the abundance of each protein. Data was log10 transformed and centered by the median method using R (version 4.2.2) for statistics analysis.

### Analysis and visualization of the O1 channel

Potential water/proton channels in Photosystem II were identified and analyzed using the Caver 3.0 PyMOL plugin (Chovancova et al. 2012) on the crystal structure with PDB ID 7N8O, essentially as in (Hussein et al. 2023). The Mn_4_​CaO_5​_ cluster and all protein residues were retained in the model. The O1 site of the oxygen-evolving complex (coordinates: x = 115.652, y = 170.4, z = 139.863) was used as the starting point for channel detection. Parameters were set to a probe radius of 0.9 Å, shell radius of 3.0 Å, shell depth of 4.0 Å, frame weighting coefficient of 1.0, and clustering threshold of 3.5 Å. The resulting channels were then inspected in PyMOL to identify lining residues and evaluate channel geometry. Candidate channels were compared and filtered based on their continuity, minimal bottleneck radius, and proximity to key catalytic residues to determine their potential relevance for water or proton transfer.

### Time resolved-membrane inlet mass spectrometry

The PSIIcc were thawed on ice and re-buffered into 50 mM MES-NaOH pH 6.5, 1 M betaine, 15 mM CaCl_2_, 15 mM MgCl_2_ using Amicon Ultra-0.5 centrifugal filters. The samples were adjusted to 0.2 mg Chl/ml, before being flashed with one saturating Xenon flash (FWHM 5 µs), and then dark adapted for a further 1 h at room temperature. Directly before transferring the PSIIcc in dim green light into the MIMS cell, 0.2 mM DCBQ was added from 100 mM stock solution in DMSO.

Substrate–water exchange rates were measured at 10^o^C employing an isotope ratio mass spectrometer (Finnigan Delta Plus XP) connected to a custom 165 µL rapid mixing reaction cell via a stainless-steel pipe that passed through a Dewar filled with liquid nitrogen. A customized solenoid-controlled syringe (based on Hamilton CR-700-50) was loaded under N_2_ atmosphere with 25 µL 97% H_2_^18^O, which was previously bubbled with nitrogen to remove the dissolved oxygen and argon. The solenoid valve was opened by a computer trigger exposing the plunger of the syringe to 6 Pa N_2_ to obtain fast (6 ms) H_2_^18^O injection and ^18^O isotope enrichment of the reaction medium. Residual O_2_ in the H_2_^18^O was estimated and removed from the data as described previously (de Lichtenberg et al. 2021a). The substrate exchange rates (k_f_ and k_s_) for the fast and slow exchanging substrate waters were determined by the global fitting of both m/z 34 and m/z 36 signals employing the following exponential functions:

1 $$\begin{aligned}\:m/z \,\space 34= \,\space&a\cdot\:\left(1-b*{e}^{-{k}_{f\cdot\:}\cdot\:t}\right)\\&+(1-a)\cdot\:(1-{e}^{-{k}_{s\cdot\:}\cdot\:t})\end{aligned}$$

2 $$\:m/z\,\space36=\left(1-{e}^{-{k}_{s\cdot\:}\cdot\:t}\right)$$

where *a* represents the percentage of fast exchanges in the m/z 34 kinetic data that is calculated using Eq. 3, where the final enrichment is $\alpha_{f} \approx 13 \space \%$ and the initial enrichment is $\alpha_{i} \approx 0.07 \space \%$:

3 $$\:a=\:\frac{{\alpha}_{f}\cdot\:\left(1-{\alpha}_{i}\right)+\left(1-{\alpha}_{f}\right)\cdot\:{\alpha\:}_{i}}{\left(1-{\alpha}_{f}\right)\cdot\:{\alpha}_{f}\cdot\:2}$$

and b is a term applied to account for the fast substrate water exchange in S_3_ in between turnover flashes for the measurements in S_2_ state, which is calculated as:

4 $$\:b=1-\left(1-{e}^{{k}_{f}^{S3}\cdot\:{t}^{S3}}\right)$$

where $$\:{k}_{f}^{S3}$$$$k_f^{S_{3}}$$denotes the rate of fast substrate exchange in S_3_ state and $$\:{t}^{S3}$$$$t^{S_{3}}$$ denotes the time spent in S_3_, which is equal 10 ms for 100 Hz turnover flashes applied for this study. No correction is needed for substrate exchange measurements in the S_3_ state, so the b-factor can simply be taken as 1. For further details of the analysis, see (Chernev et al. 2024).

### In vivo variable fluorescence measurements

Flash-induced changes in the chlorophyll fluorescence yield (variable fluorescence decay kinetics) were measured as with the FL3300 dual-modulation fluorometer (Photon System Instruments, Czech Republic) in the 150 µs–100 s time range. Actinic flash duration was 30 µs and 8 measuring flashes per decade were applied in a logarithmic scale with a duration of 2.5 µs (Volgusheva et al. 2013, 2016).

The wild type and mutant *Synechocystis* cultures were grown in BG11 medium in 6-well plates under 30 µE light. The cell cultures are collected and diluted into 5 µg Chl ml^− 1^, before an hour of dark adaptation. For each measurement, a 1.5 ml aliquot is taken from the same stock, dark-adapted for 5 min before the fluorescence decay kinetics were measured. The measurements were done in the absence or presence of 25 µM DCMU added from the 10 mM stock solution. All measurements were performed in triplicates. The fluorescence decay kinetics are analyzed as convolution of three exponential decay components (Mamedov et al. 2000; Volgusheva et al. 2016), using the following model:

5 $$\begin{aligned}F_V^{norm}\left( t \right) =\,& {A_0} + {A_1}{e^{\left( {{\raise0.7ex\hbox{${ - t}$} \!\mathord{\left/{\vphantom {{ - t} {{\tau _1}}}}\right.\kern-\nulldelimiterspace}\!\lower0.7ex\hbox{${{\tau _1}}$}}} \right)}}\\& + {A_2}{e^{\left( {{\raise0.7ex\hbox{${ - t}$} \!\mathord{\left/{\vphantom {{ - t} {{\tau _2}}}}\right.\kern-\nulldelimiterspace}\!\lower0.7ex\hbox{${{\tau _2}}$}}} \right)}} + {A_3}{e^{\left( {{\raise0.7ex\hbox{${ - t}$} \!\mathord{\left/{\vphantom {{ - t} {{\tau _3}}}}\right.\kern-\nulldelimiterspace}\!\lower0.7ex\hbox{${{\tau _3}}$}}} \right)}}\end{aligned}$$

where the normalized variable fluorescence is obtained as:

6 $$\:{F}_{V}^{norm}\left(t\right)=\frac{{F}_{v}\left(t\right)}{{F}_{max}-{F}_{0}}.$$

### Electron paramagnetic resonance spectroscopy

PSIIcc were thawed on ice, washed into 50 mM MES-NaOH (pH 6.5), 1 M betaine, 15 mM CaCl_2_, 15 mM MgCl_2_ buffer using an Amicon Ultra-0.5 with a cutoff of 100 kDa and concentrated to a final Chl concentration of 1.5–2.5 mg/ml. Traces of dissolved Mn^2+^ were complexed by 1 mM ethylenediaminetetraacetic acid (EDTA) to suppress the EPR six-line signal. For full oxidation of Y_D_, EPR samples were exposed for one minute to room light while rotating, followed by 90 min of dark adaptation at room temperature. These dark-adapted samples were frozen in a dry ice/ethanol bath and transferred to liquid N_2_ for storage before the measurements.

Low temperature continuous wave EPR measurements were performed with an X-band EMX Micro spectrometer equipped with a EMX Premium bridge and ER4119HS resonator (Bruker BioSpin GmbH, Germany). The system was fitted with an Oxford 900 cryostat and an ITC-503 temperature controller from Oxford instruments Ltd., UK. The S_2_ state multiline (ML) signal was induced by illumination at 200 K for 6 min.

## Results

### O1 channel bottleneck characteristics in *Synechocystis*

The O1 channel network has been shown to include two branches in *T. vestitus*, O1A and O1B. Of these, we consider only the O1B branch relevant to our analysis of *Synechocystis* PSII, as the O1A branch leads to the CP43 domain, where access to the lumen is restricted by the thylakoid lipid molecule digalactosyl diacylglycerol (DGDG), confirming a previous analysis (Hussein et al. 2023). For simplicity, we refer to the O1B branch as the O1 channel.

**Fig. 2:**
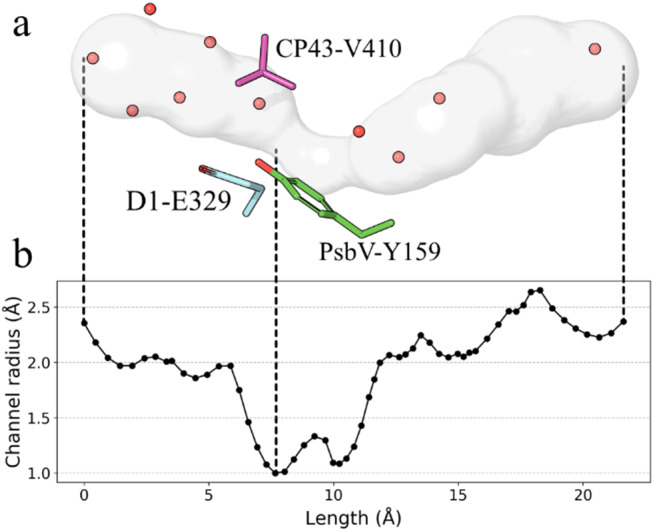
The profile of the calculated O1 channel. (**a**) The channel model is depicted as the grey surface leading from the O1 bridge (left) to the lumen (right). Red spheres are showing the molecules aligned in the channel cavity. The bottleneck residues are displayed. Structures based on PDB entry 7N8O. (**b**) The radius of the channel cavity versus the length from the O1 bridge as the starting point. Dashed lines between (**a**) and (**b**) are drawn to indicate specific locations; start point, bottleneck, end point from left to right

Our analysis reveals that the O1 channel cavity extends approximately 20 Å from the Mn_4_CaO_5_ cluster to the lumen, with its bottleneck defined by residues from D1, CP43, and PsbV, as shown in Fig. 2. The channel radius narrows to about 1 Å near PsbV-159, which has been proposed to obstruct water molecule passage through this pathway. This radius is comparable to the bottleneck radii of O1 channels in Photosystem II core complexes (PSIIcc) from other species, which range from 0.9 Å to 1.1 Å (Hussein et al. 2023), suggesting that it does not introduce any additional constraints, regardless of the tyrosine residue present in *Synechocystis* PSIIcc.

### Substrate exchange kinetics

**Fig. 3:**
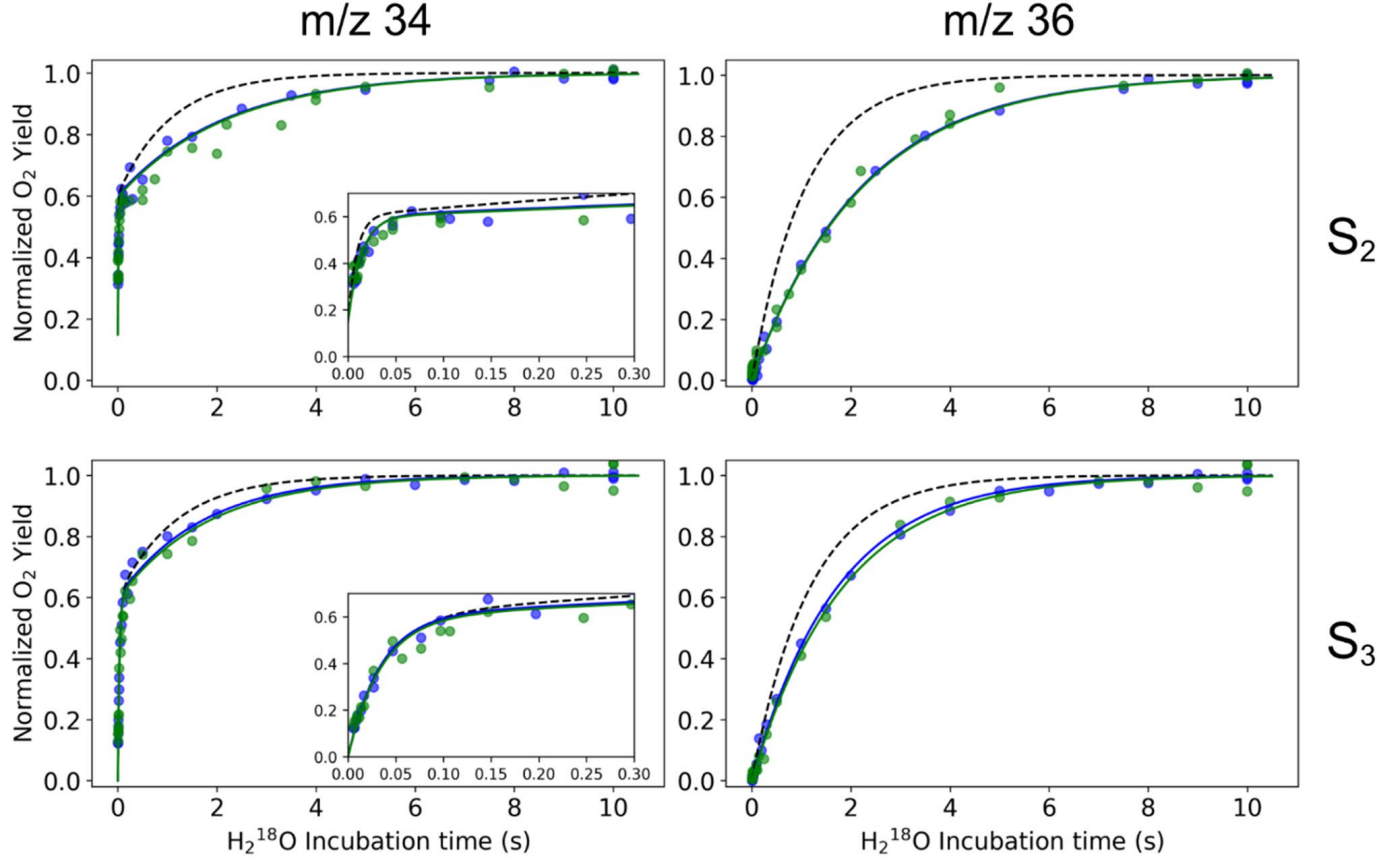
Substrate water exchange kinetics of PSII core complexes isolated from *Synechocystis*. Data (symbols) and fits (solid lines) for the site directed mutants D1-E329F and D1-E329L are shown in blue and green, respectively, while the dashed black lines show the fit obtained for WT PSII data (see SI Figure S2 for the WT data). The titles m/z 34 and m/z 36 denote the single labeled oxygen (^18,16^O_2_) and double labeled oxygen (^18,18^O_2_), respectively. The insets in m/z 34 graphs show a detailed view of the fast phase of substrate water exchange reflecting W_f_ exchange. The data recorded at 10 °C and pH 6.5

**Fig. 4:**
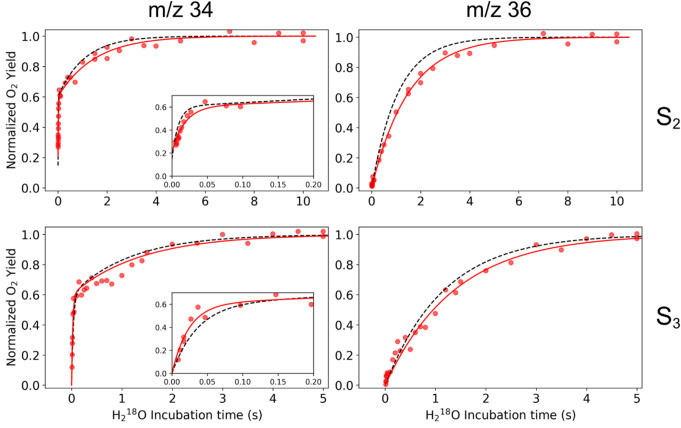
Substrate water exchange kinetics of PSII core complexes isolated from *Synechocystis*. Results (symbols) and fits (solid lines) for the site directed mutant CP43-V410S is shown in red, while the fit obtained for WT PSII is shown as dashed black lines (see SI Figure S2 for the WT data). The titles m/z 34 and m/z 36 denote the single labeled oxygen (^18,16^O_2_) and double labeled oxygen (^18,18^O_2_), respectively. The insets in m/z 34 graphs show an expansion of the fast phase of substrate water exchange reflecting W_f_ exchange. The data were recorded at 10 °C and pH 6.5. Note the change in the time scale between top and bottom panels

The substrate water exchange kinetics of PSIIcc from wild type (WT), D1-E329F, D1-E329L, and CP43-V410S Synechocystis were investigated in the S_2_ and S_3_ states at 10 °C and pH 6.5. For all variants, the time dependence of the m/z 34 signal displayed a biphasic exponential rise in both states, indicating the presence of two substrate waters with kinetically distinct exchange rates: a fast-exchanging water (W_f_) and a slow-exchanging water (W_s_), consistent with previous reports (reviewed in Cox and Messinger 2013), see Figures 3 and 4. The monophasic rise of the m/z = 36 signal, which is constrained by the W_s_ exchange rate, confirms the absence of sample heterogeneity, in line with the similar protein composition of WT and mutant PSIIcc (see Figure S1). The exchange rates, k_f_ (for W_f_) and k_s_, (for W_s_), were determined by simultaneously fitting the m/z 34 and m/z 36 traces using equations 1-4. The rates are summarized in Table 1.

**Table 1:** Substrate water exchange rates in the S2 and S3 states of PSII core complexes purified from wild-type (WT), D1-E329F, D1-E329L and CP43-V410S mutants of *Synechocystis*. The experimental data and fits are shown in Figures 4 and 5. Errors were determined by the distribution of fitted parameters obtained by a jackknife resampling protocol (Efron and Tibshirani 1994) (see SI Figure S3)

Samples	W_f_ exchange		W_s_ exchange	
	S_2_	S_3_	S_2_	S_3_
	k_f_ (s^− 1^)	k_f_ (s^− 1^)	k_s_ (s^− 1^)	k_s_ (s^− 1^)
WT	111 ± 8	29 ± 2	0.93 ± 0.03	0.85 ± 0.03
D1-E329F	68 ± 4	29 ± 2	0.45 ± 0.02	0.58 ± 0.02
D1-E329L	72 ± 7	29 ± 3	0.45 ± 0.03	0.55 ± 0.03
CP43-V410S	55 ± 4	42 ± 5	0.67 ± 0.03	0.73 ± 0.03

The exchange rates for WT PSII obtained here (Figure S2 and Table 1; dashed lines in Figs. 3 and 4) differ slightly from those reported previously (de Lichtenberg, et al. 2021a, b). The discrepancy in the k_f_ values in S_2_ arises from the additional correction applied for non-instant mixing (Table S1; see also (Chernev et al. 2024).

Our investigation resulted in nearly identical substrate exchange kinetics for both D1-E329F and D1-E329L mutants in the S_2_ and S_3_ states (see Fig. 3). W_f_ exchange rates are reduced by 35–40% with the introduction of these hydrophobic and uncharged residues instead of the hydrophilic and negatively charged glutamic acid in WT PSIIcc (Table 1). However, no effect was observed in W_f_ exchange rates in S_3_, in line with the proposed change in the ligation mode of the fast-exchanging substrate water during the S_2_→S_3_ transition through which W_f_ is ligated to Mn1^IV^ in the S_3_ state and thus its exchange no longer limited by the diffusion of water molecules into the active site (de Lichtenberg et al. 2021b).

Contrary to our expectation, studied modifications in the outer sphere of the active site had a significant impact on the W_s_ exchange kinetics. Our analysis revealed that the W_s_ exchange in the D1-E329 mutants is approximately 50% slower in S_2_ and 30% slower in S_3_ relative to WT-PSII (Table 1). This consistent effect marks the importance of the protein-water network integrity in the O1 channel for an efficient substrate exchange mechanism. Possible structural implications of these mutations are discussed below.

**Fig. 5:**
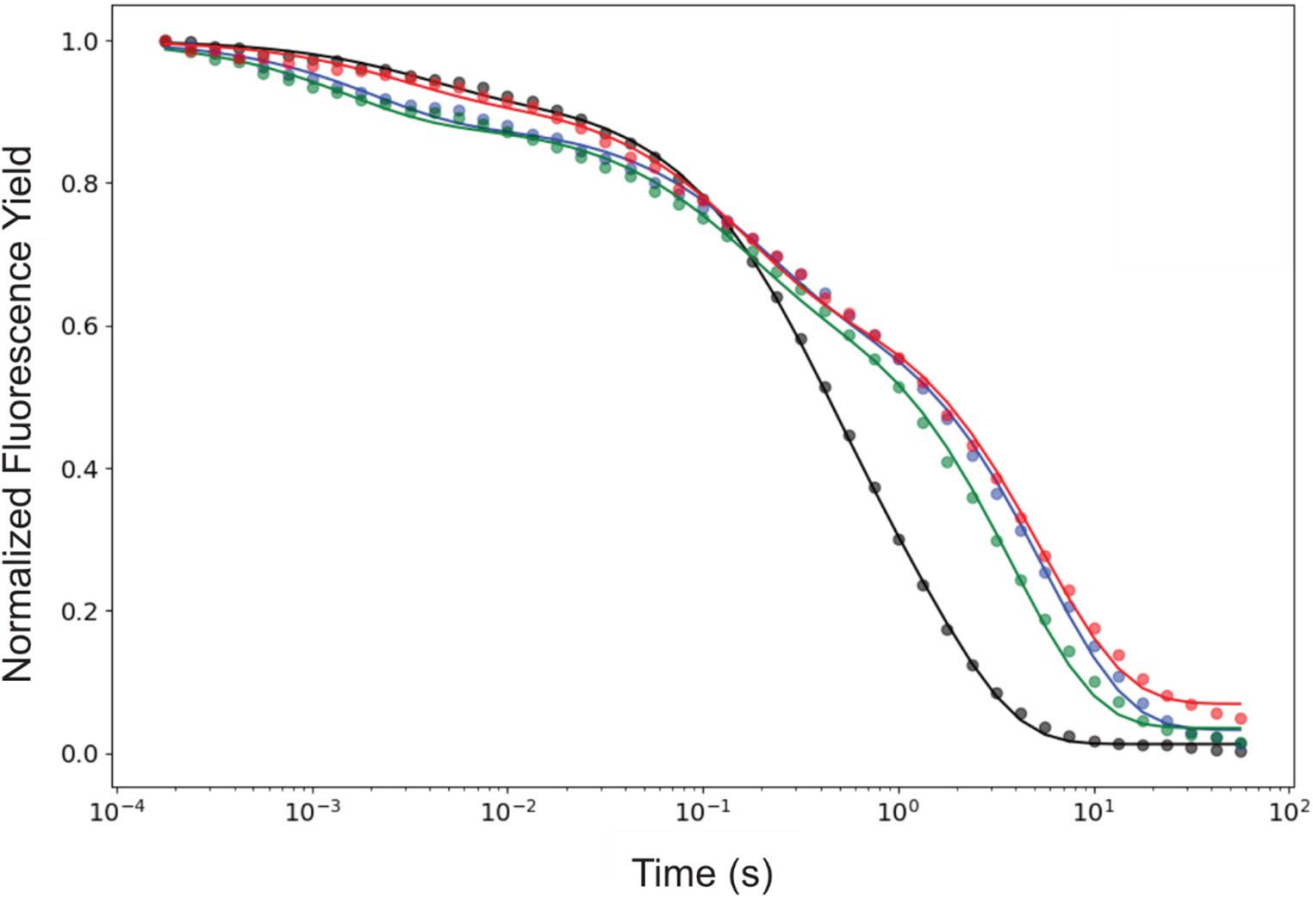
Normalized variable fluorescence decay kinetics recorded after a single actinic flash applied on whole cells in the presence of 25 µM DCMU. The lines denote the three exponential fit model optimized for the datasets shown with spheres. WT, E329F, E329L and V410S are shown with colors; black, blue, green and red, respectively

In the S_2_ state, the W_f_ exchange rate is reduced by 50% in the CP43-V410S modification, while the same mutation increased the rate of W_f_ exchange in the S_3_ state by 40% compared to WT (insets in Fig. 5; Table 1), eventually leading to comparable exchange rates in both S states. The CP43-V410 residue connects to the Mn_4_CaO_5_ via 5 water molecules arranged as water wheel residing in the O1channel (see Fig. 1c). Analysis of current structural studies highlighting the dynamics in the region is crucial to understand possible rate limitations enforced by CP43-V410S modification on the W_f_ exchange.

Like the D1-E329 mutations, CP43-V410S slows the W_s_ exchange rate by approximately 30% in S_2_, but in contrast to the two other mutations, only marginally (15%) in the S_3_ state (Table 1). To understand how this outer sphere modifications can act on the chemical exchange of W_f_ (O_X_ in the S_3_ state) and W_s_ (O5; in the S_2_ and S_3_ states), we need to carefully analyze the rate limiting steps of substrate exchange in PSII (see Discussion). Independent of the precise mechanisms, the results obtained here show that alterations as far as 10 Å away from the Mn_4_CaO_5_ cluster affect the catalytic site and thus these remote amino acids may be considered part of the OEC.

### Fluorescence decay measurements

We investigated the charge recombination kinetics in WT, D1-E329F, D1-E329L and V410S mutants using variable fluorescence spectroscopy. The variable fluorescence decay upon a single actinic flash applied in the S_1_ state in absence of an inhibitor showed no significant effect across all samples, indicating that the forward electron transfer is not impaired by the mutations (see Figure S4) (Mamedov et al. 2000). This observation demonstrates that the modifications in the current investigation had no impact on the energetics of redox co-factors on the acceptor site of PSII.

The electron transfer to the Q_B_ site is effectively blocked in the presence of DCMU, allowing the study of the charge recombination kinetics from Q_A_^●−^ with the PSII donor site (Fig. 5). The deconvolution of recombination kinetics in WT and mutant *Synechocystis* cells is given in Table 2. The fast phase arises from the recombination with P680^●+^ in PSII centers not active in O_2_ evolution and is thus not relevant here. The middle and slow phases describe the charge transfer from Q_A_^●−^ back to Y_Z_^●^ and the S_2_ state, respectively (Mamedov et al. 2000). The population of these phases reflects the S_1_Y_Z_^●^ ⇆ S_2_Y_Z_ equilibrium, which is pH dependent and thus the electron transfer is coupled to a proton transfer (Gasanov et al. 2007; Volgusheva et al. 2016). The half-times and amplitudes obtained by deconvolution of the fluorescence decay for WT *Synechocystis* are consistent with the literature (Nixon and Diner 1992; Vass et al. 1999).

**Table 2:** Flash-induced fluorescence decay half-times and amplitudes (in parentheses) for wild type and mutant *Synechocystis* cells in the presence of 25 ΜM DCMU. The reported uncertainties are taken as the square roots of the diagonal elements of the parameter covariance matrix

Samples	Fast phase		Middle phase		Slow phase
	τ_1_ (ms) [amp (%)]		τ_2_ (ms) [amp (%)]		τ_3_ (s) [amp (%)]
WT	3.5 ± 0.4 (7%)		289 ± 22 (37%)		1.5 ± 0.1 (54%)
D1-E329F	2.0 ± 0.3 (12%)		185 ± 19 (23%)		5.5 ± 0.2 (62%)
D1-E329L	1.5 ± 0.2 (12%)		140 ± 15 (22%)		3.8 ± 0.2 (63%)
CP43-V410S	2.7 ± 0.7 (8%)		133 ± 13 (27%)		5.4 ± 0.2 (59%)

With respect to the WT, the slow fluorescence decay kinetics of the D1-E329F, D1-E329L, and V410S mutants revealed a significant slowing of the slow phase, with half-times approximately 3–4 times longer and of slightly increased amplitude, indicating a stabilization of the S_2_Y_Z_ state in all mutants. In line with this, the middle phase was found to be slightly faster and decreased in population compared to WT (see Table 2; Fig. 5). These observations suggest that all three mutations altered the H-bonding network, which in turn stabilized the S_2_ state and destabilized Y_Z_^●^.

### EPR spectroscopy

**Fig. 6:**
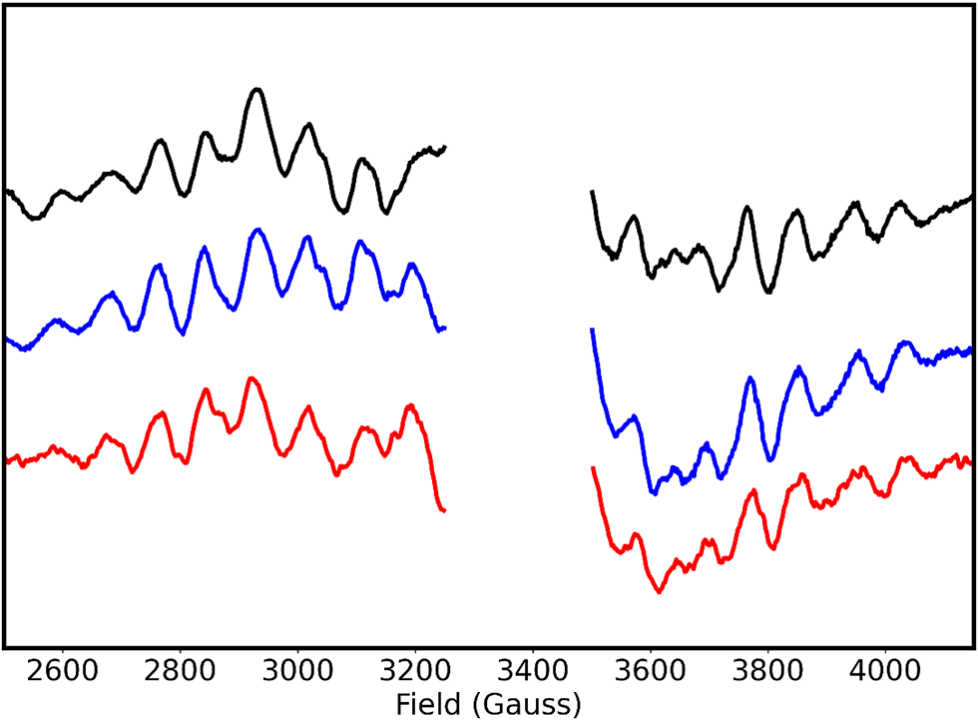
Light-minus-dark S_2_ state multiline EPR signals obtained for WT, D1-E329F and CP43-V410S PSIIcc by illumination at 200 K. The spectra are normalized with respect to the chlorophyll concentrations. WT, E329F and V410S are shown with colors; black, blue and red, respectively. EPR conditions: microwave frequency: 9.38 GHz, microwave power: 20 mW, modulation amplitude: 20 G, temperature: 7 K

It was recently shown that the heterogeneity in the Mn_4_CaO_5_ cluster conformations in PSII affects the substrate exchange kinetics; specifically it was demonstrated that the S_2_^HS^ conformation promotes faster W_S_ exchange (de Lichtenberg et al. 2020; Chernev et al. 2024). Although the effect on the slow water exchange kinetic in this earlier study was opposite to that observed here, we investigated possible effects of the D1-E329F and CP43-V410S mutations on the Mn_4_CaO_5_ cluster by measuring the S_2_ state EPR multiline signal that arises from S_2_^LS^ conformation. As seen in Fig. 6, the signals from the WT and mutants are highly similar both in signal shape and intensity, indicating that the structure of the Mn_4_CaO_5_ cluster is not altered by the mutations.

## Discussion

The present study was designed to (i) test if the O1 channel network facilitates water access to the Mn_4_CaO_5_ cluster and to (ii) gain insight into how the protein-water-cofactor network of the OEC regulates water delivery and exchange. Below, we discuss various aspects of our results to address these research questions.

### The O1 channel regulates water access to the Mn_4_CaO_5_ cluster

On the basis of a previously obtained CryoEM structure, it was concluded that in *Synechocystis* the O1 channel network is blocked by the PsbV-Y159 residue (Gisriel et al. 2022). However, our evaluation of the published data using CAVER 3.0 (Chovancova et al. 2012) revealed a water filled channel reaching from the lumen to the O1 bridge in the Mn_4_CaO_5_ cluster with a minimum radius of 1.0 Å (Figs. 1c and 2), comparable to the bottleneck radius for O1 channel for *T. vestitus* PSIIcc estimated to be 1.1 Å (Hussein et al. 2023). This suggests that the existence PsbV-Y159 residue in *Synechocystis* PSIIcc poses no blockage to access of water molecules through the O1 channel. Our conclusion is further supported by a previous study succesfully tracking a continuous ROS release pathway from the O1 site to the lumen in *Synechocystis* PSII (Weisz et al. 2017).

In this study, the exchange of W_f_ in the S_2_ state is a sensitive probe for water access to the Mn_4_CaO_5_ cluster, since it reports on the isotopic equilibration of the inner water pool of the OEC with bulk water (de Lichtenberg et al. 2021b; Chernev et al. 2024). All three mutations in the bottleneck caused a retardation of W_f_ exchange in the S_2_ state, providing conclusive evidence for water access through the O1 channel. The invariance or slight increase in W_f_ exchange rate in the S_3_ state is consistent with W_f_ binding to Mn(IV) during the S_2_→S_3_ transition, so that its exchange is slower (it requires electron back donation from Y_Z_; see below) and its exchange is no longer limited by the rate of isotopic equilibration of the OEC’s water pool.

The present data does not allow us to derive the precise mechanism of how the bottleneck mutants constrain water access and thereby slow the isotopic equilibration of the OEC’s water pool; however, it is likely that both steric as well as dynamic effects contribute. Thereby, these data are consistent with previous proposals of controlled water access to the Mn_4_CaO_5_ cluster that prevents an excess number of water molecules in the catalytic site that could cause unwanted side reactions (Tso et al. 1991; Wydrzynski et al. 1996; Ho and Styring 2008). In the present view, regulation of water access by gates in the three channels leading the OEC appears to be required both for preventing manganese oxide formation and for maintaining an ordered yet dynamic H-bonding network within the OEC.

These ideas are strongly supported by structural snapshots obtained by serial crystallography experiments during the S_2_→S_3_ transition, which show that the D1-E189, D1-E329 and CP43-V410 residues move in tandem with the insertion of O_X_ (Ibrahim et al. 2020; Hussein et al. 2021; Li et al. 2024). Similar movements of amino acids were recently also associated with water binding during S_0_ formation in the and S_3_→S_4_→S_0_ transitions (Bhowmick et al. 2023).

Despite being remote from the Mn_4_CaO_5_ cluster, the three site specific mutations also affected the W_S_ exchange in the S_2_ and S_3_ states, and, for CP43-V410S, also the fast exchange in the S_3_ state, which are all slower than the fast exchange in the S_2_ state and limited by chemical exchange. The detailed discussion below analyzes how protein-water-cofactor interactions, including the pentameric water wheel of the O1 channel, can regulate water insertion and exchange at the Mn_4_CaO_5_(-O_X_) cluster.

### Effects of the bottleneck mutations on the OEC

Our O1 channel bottleneck modifications were introduced at residues distant from the Mn_4_CaO_5_ cluster to minimize direct effects on its electronic structure and conformation. In our substrate exchange data, the monophasic rise in the m/z 36 traces was preserved in all cases, showing no indication for Mn_4_CaO_5_ cluster heterogeneity (see Figs. 3 and 4). In addition, the X-band EPR spectra of WT and mutant PSII show S_2_ multiline signals of comparable signal shape and intensity (Fig. 6), confirming that the conformation and electronic structure of the OEC are not changed. Thus, the effects of the three mutants on the water exchange must be due to alterations in the protein-water-cofactor network and its dynamics.

The site-specific modifications in our current study are connected to the Mn_4_CaO_5_ cluster via the water wheel (Fig. 1a). Thus, we propose that the D1-E329F, D1-E329L and CP43-V410S mutations affect the structure or dynamics of the water wheel, which in turn alters the water exchange kinetics. In line with this proposal, it was previously shown that modifications of the D1-E329 residue eliminate carbonyl vibrations in the FTIR spectra, specifically the 1747 cm⁻¹ feature, indicating its involvement in long ranged H-bonded networks that could pose a constraint for proton transfer during the S-state transitions (Service et al. 2010; Kim and Debus 2020).

### Rate limitations during O5 and O_X_ exchange

**Scheme 1:**
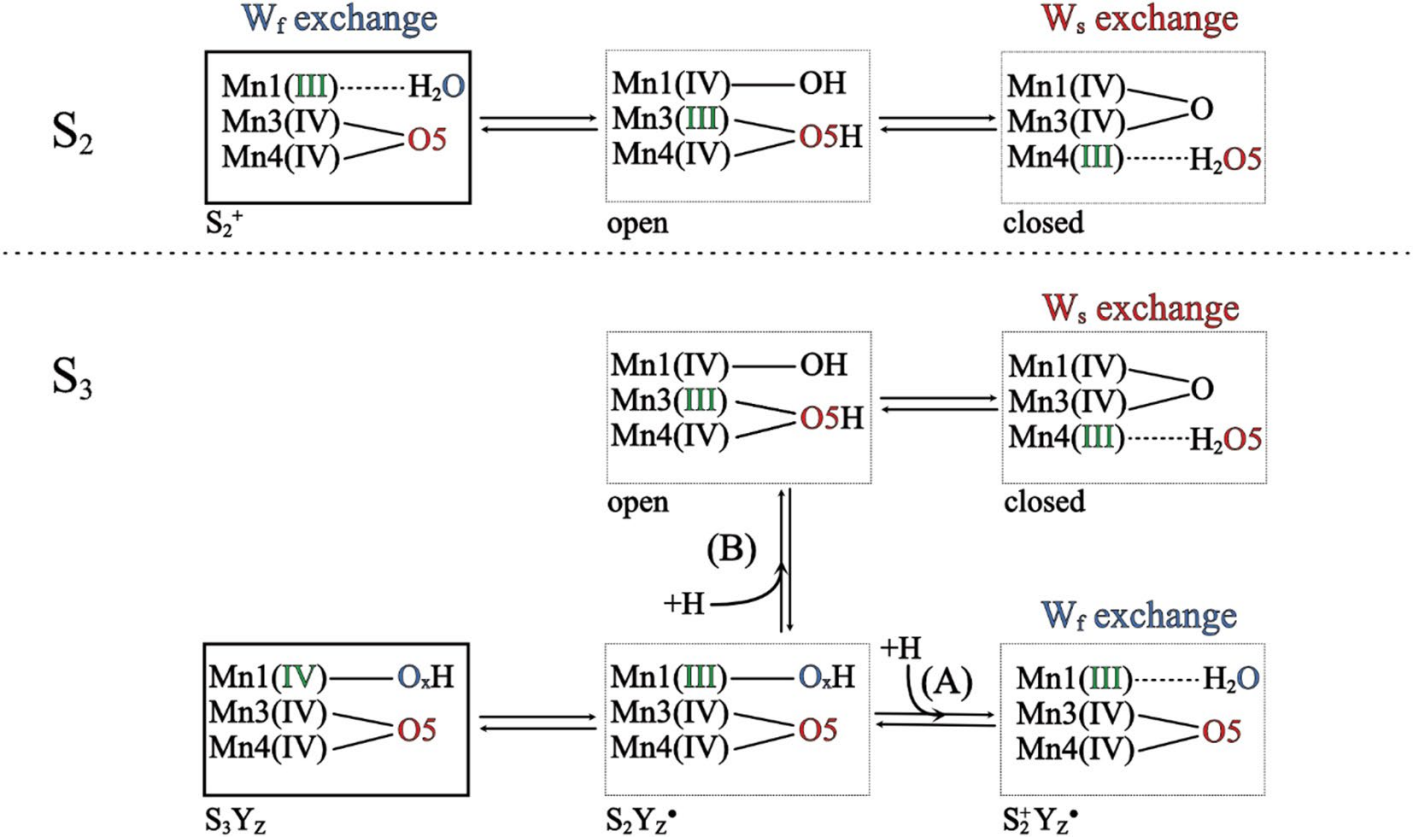
Simplified and expanded representation of the substrate exchange mechanism in S_2_ and S_3_ states proposed by Siegbahn (Siegbahn, 2013). The red and blue oxygen atoms denote W_s_ and W_f_, respectively. The upper row is showing the exchange in the S_2_ state, while the lower row is a possible exchange mechanism in the S_3_ state. Paths A and B denote protonation steps needed to initiate W_f_ and W_s_ exchange, respectively. The association of O5 and O_X_ with Ca is not shown for simplicity

Before starting the detailed analysis of the observed effects on substrate water exchange kinetics it is necessary to differentiate between substrate water exchange and water insertion during S_2_→S_3_ and S_3_→S_4_→S_0_ transitions. Water binding during the S_2_→S_3_ transition (τ ≈ 350 µs) (Suga et al. 2019; Ibrahim et al. 2020; Okamoto et al. 2021) and water insertion during S_3_→S_4_→S_0_ transition (τ ≈ 2000 µs) (Bhowmick et al. 2023) are light-induced and much faster processes than the substrate water exchange (τ > 10 ms). The former requires the binding of just one well positioned water molecule, while the latter involves the isotopic equilibration of all exchangeable water molecules in the OEC (W_f_ in S_2_) as well as the chemical exchange of Mn-bound, deprotonated water species (O5 and O_X_). Nevertheless, according to Scheme 1, the two processes are connected since W_s_ exchange in S_2_ requires O_X_ binding and, in the S_3_ state, W_f_ is O_X_ (Scheme 1; modified after Siegbahn 2013).

The ^18^O exchange of substrate water molecules are governed by a series of complex dynamics. It was previously argued that the aqua ligands to Mn(IV) species are exchange inert while the exchange of water ligands of Mn(III) are possible (Hillier and Wydrzynski 2001, 2008). Additionally, oxo species must be protonated before they can be exchanged with the bulk water (Lundberg et al. 2003).

The slow exchanging water, W_s_, can readily be observed from S_0_ to S_3_ (Messinger et al. 1995; Hillier and Wydrzynski 2008; Cox and Messinger 2013), and has been identified as the central µ_3_-oxo bridge O5 that is connected to Ca, Mn3 and Mn4, where both Mn ions are in oxidation state IV in S_2_ (Messinger 2004; Siegbahn 2009, 2013; Rapatskiy et al. 2012; de Lichtenberg et al. 2024). In the substrate water exchange mechanism proposed by Siegbahn (Scheme 1; Siegbahn 2013), the exchange of O5 (W_s_) is initiated, in the S_2_ state, by water binding to Mn1, which is in the formal oxidation state III. A sequence of proton and electron transfers as well as an open-closed type conformational change are needed to sequentially protonate the O5-site and to expose it, fully protonated, for exchange in a terminal position at Mn4(III). The first electron transfer from Mn1 to Mn3 is coupled with the first protonation, which was described as the rate limiting step with E_a_ = 17.6 kcal/mol, matching with the experimental value (Hillier and Wydrzynski 2000). In the S_3_ state, all Mn ions are in the formal oxidation state IV (Cox et al. 2014), so that the Mn_4_CaO_5_-O_X_ cluster needs to be reduced to allow water exchange. The most reasonable reductant is Y_Z_, yielding S_2_Y_Z_^●^ and Mn1(III) (Geijer et al. 2001; Rappaport and Diner 2008). In comparison to the S_2_ state, the Mn_4_CaO_5_-O_X_ cluster needs to receive an extra proton to initiate W_s_ exchange. Since O_X_ is already in place, the exchange may follow either route A or B. It is of note, that all steps involve proton coupled electron transfer (PCET) steps (see Scheme 1).

### W_s_ is slowed due to inefficiencies in water insertion and/or proton transfer

In the S_2_ state, modifications on D1-E329 side chain into phenylalanine and leucine are imposing 2-fold slower exchange of O_X_ (W_S_). According to Scheme 1, the primary rate limitation is the binding of a water molecule to the Mn1 ion, which requires a PCET step. The electron transfer is essentially a translocation of charge within the Mn_4_CaO_5_ cluster, which we do not anticipate being affected by the D1-E329 side chain mutations, due to the similar S_2_ multiline EPR spectra (Fig. 6). On the other hand, the variable fluorescence data suggest that D1-E329F and D1-E329L are altering the H-bonding network. Since water binding to Mn1 requires proton transfer from water to O5 that possibly occurs via the H-bonding network (Shen 2015; Nagao et al. 2017; Okamoto et al. 2021; Greife et al. 2023), this rate limiting process may be slowed by the mutant-induced alterations of the H-bonding network.

However, we cannot exclude a more direct effect; the D1-E329 headgroup orientation was shown to vary among species (Hussein et al. 2023) and to be located, in *Synechocystis*, close to D1-E189 and D1-H332 (Fig. 1c). Thus, the D1-E329F and D1-E329L mutations may modify the position and/or flexibility of the D1-H332 and D1-E189 side chains, which could affect their known movements during water insertion (Hussein et al. 2021; Isobe et al. 2024) and thereby slow down water binding and O5 exchange. Indeed, in a previous study, the D1-H332Q mutation was shown to slow W_s_ exchange 2-fold in the S_3_ state (Sugiura et al. 2009).

For the CP43-V410S mutant we observed a 30% slower W_s_ exchange rate in the S_2_ state when compared to WT; thus, it causes a similar but less pronounced effect than the two D1-E329 mutants. In this case, the effect cannot be attributed to an interaction with first-sphere residues as CP43-V410S is 10 Å away. The modification of the valine to a serine introduces an additional H-bond partner in the bottleneck, which likely disturbs the H-bonding network including the water wheel. This could lead to delays or inefficiencies in proton translocation, as the structured pathway becomes disordered, eventually introducing an increased energy barrier for water insertion.

In the S_3_ state, the effect of the CP43-V410S mutation on W_s_ exchange is even smaller, only a 15% slowing is observed. In this state, O_X_ is already present (path B in Scheme 1), which may explain the smaller effect. Thus, this observation can instead be ascribed to effects on the required protonation of O5.

Interestingly, in CP43-V410S PSIIcc, the rate of W_f_ exchange in the S_3_ state is 40% faster than in WT PSIIcc. Since a previous study reports that CP43-V410 rotates 120° during the S_2_ to S_3_ transition to accommodate a new water molecule of in *T. vestitus* PSII structure (Li et al. 2024), we hypothesize that an organizational change is needed to both allow faster water entry and O_x_ insertion. If so, introduction of the hydrophilic serine side chain may emulate the required change and thereby decrease the energy barrier for the protonation and reinsertion of labeled O_X_.

These interpretations are in line with our variable fluorescence data, which indicate that the O1 channel mutations modify both the S_2_ state stability and the H-bonding network required for efficient stabilization of Y_Z_^●^, likely by affecting the Y_Z_-H190-N298 triad (Mamedov et al. 1998) and/or connected groups of water molecules that may act as proton storage sites (Chrysina et al. 2019; Okamoto et al. 2021). In other enzymes, similar planar water pentamers were shown to be structurally rigid, yet able to perform flip rearrangement allowing internal proton exchange (Liu et al. 1996; Xu et al. 2020) or to facilitate proton storage (Maurer and Oostenbrink 2019; Gerwert et al. 2014).

## Conclusion

Water splitting by the Mn_4_CaO_5_ cluster in PSII requires a highly intricate interplay between the base metal cofactor and the protein-water network. Our current results support the hypothesis that the O1 channel is the primary water uptake pathway, in contrast to previous claims that this pathway is closed in *Synechocystis*. This study also highlights the important role of the pentameric water wheel as proton sink, which facilitates rapid protonation/deprotonation needed for the binding and exchange of substrate water molecules. Our study is an example of how protein-water-cofactor interactions, and the associated H-bonding networks tune the properties of the Mn_4_CaO_5_ cluster, thereby enabling water oxidation. All data are consistent with O5 and O_X_ being the substrate water molecules forming the O-O bond, thereby supporting O_2_ formation in PSII via oxo-oxyl radical coupling.

## Electronic supplementary material

Below is the link to the electronic supplementary material.

Supplementary Material 1

## Data Availability

The authors declare that the data supporting the findings of this study are available within the paper and its Supplementary Information files. Should any raw data files be needed in another format they are available from the corresponding author upon reasonable request. The mass spectrometry proteomics data have been deposited to the ProteomeXchange Consortium via the PRIDE partner repository with the dataset identifier PXD061921.

## References

[CR1] Bhowmick A, Hussein R, Bogacz I et al (2023) Structural evidence for intermediates during O_2_ formation in photosystem II. Nature 617:629–636. 10.1038/s41586-023-06038-z 37138085 10.1038/s41586-023-06038-z PMC10191843

[CR2] Boussac A, Ugur I, Marion A et al (2018) The low spin - high spin equilibrium in the S_2_-state of the water oxidizing enzyme. Biochim Biophys Acta Bioenerg 1859:342–356. 10.1016/j.bbabio.2018.02.010 29499187 10.1016/j.bbabio.2018.02.010

[CR3] Bovi D, Narzi D, Guidoni L (2013) The S_2_ state of the oxygen-evolving complex of photosystem II explored by QM/MM dynamics: spin surfaces and metastable states suggest a reaction path towards the S_3_ state. Angew Chem Int Ed 52:11744–11749. 10.1002/anie.201306667 10.1002/anie.201306667 PMC3952239 24115467

[CR4] Cheah MH, Zhang M, Shevela D et al (2020) Assessment of the manganese cluster’s oxidation state via photoactivation of photosystem II microcrystals. Proc Natl Acad Sci U S A 117:141–145. 10.1073/pnas.1915879117 31848244 10.1073/pnas.1915879117 PMC6955365

[CR5] Chernev P, Aydin AO, Messinger J (2024) On the simulation and interpretation of substrate-water exchange experiments in photosynthetic water oxidation. Photosynth Res 162:413–426. 10.1007/s11120-024-01084-8 38512410 10.1007/s11120-024-01084-8 PMC11639282

[CR6] Chovancova E, Pavelka A, Benes P et al (2012) CAVER 3.0: A tool for the analysis of transport pathways in dynamic protein structures. PLOS Comput Biol 8:e1002708. 10.1371/journal.pcbi.1002708 23093919 10.1371/journal.pcbi.1002708 PMC3475669

[CR7] Chrysina M, de Mendonça Silva JC, Zahariou G et al (2019) Proton translocation via tautomerization of Asn298 during the S_2_–S_3_ state transition in the oxygen-evolving complex of photosystem II. J Phys Chem B 123:3068–3078. 10.1021/acs.jpcb.9b02317 30888175 10.1021/acs.jpcb.9b02317 PMC6727346

[CR8] Corry TA, O’Malley PJ (2019) Proton isomers rationalize the high- and low-spin forms of the S_2_ state intermediate in the water-oxidizing reaction of photosystem II. J Phys Chem Lett 10:5226–5230. 10.1021/acs.jpclett.9b01372 31429574 10.1021/acs.jpclett.9b01372

[CR9] Cox N, Messinger J (2013) Reflections on substrate water and dioxygen formation. Biochim Biophys Acta BBA - Bioenerg 1827:1020–1030. 10.1016/j.bbabio.2013.01.013 10.1016/j.bbabio.2013.01.013 23380392

[CR10] Cox N, Retegan M, Neese F et al (2014) Electronic structure of the oxygen-evolving complex in photosystem II prior to O-O bond formation. Science 345:804–808. 10.1126/science.1254910 25124437 10.1126/science.1254910

[CR11] Dau H, Haumann M (2008) The manganese complex of photosystem II in its reaction cycle—Basic framework and possible realization at the atomic level. Coord Chem Rev 252:273–295. 10.1016/j.ccr.2007.09.001

[CR12] de Lichtenberg C, Avramov AP, Zhang M et al (2021a) The D1-V185N mutation alters substrate water exchange by stabilizing alternative structures of the Mn_4_Ca-cluster in photosystem II. Biochim Biophys Acta BBA - Bioenerg 1862:148319. 10.1016/j.bbabio.2020.148319 10.1016/j.bbabio.2020.148319 32979346

[CR13] de Lichtenberg C, Kim CJ, Chernev P et al (2021b) The exchange of the fast substrate water in the S_2_ state of photosystem II is limited by diffusion of bulk water through channels– implications for the water oxidation mechanism. Chem Sci 12:12763–12775. 10.1039/D1SC02265B 34703563 10.1039/d1sc02265b PMC8494045

[CR14] de Lichtenberg C, Messinger J (2020) Substrate water exchange in the S_2_ state of photosystem II is dependent on the conformation of the Mn 4 Ca cluster. Phys Chem Chem Phys 22:12894–12908. 10.1039/D0CP01380C 32373850 10.1039/d0cp01380c

[CR15] de Lichtenberg C, Rapatskiy L, Reus M et al (2024) Assignment of the slowly exchanging substrate water of Nature’s water-splitting cofactor. Proc Natl Acad Sci 121:e2319374121. 10.1073/pnas.2319374121 38437550 10.1073/pnas.2319374121 PMC10945779

[CR86] Efron B, Tibshirani RJ (1994) An Introduction to the Bootstrap. Chapman and Hall/CRC, New York 10.1201/9780429246593

[CR16] Elhai J, Wolk CP (1988) Conjugal transfer of DNA to cyanobacteria. In: Methods in Enzymology. Academic Press, pp 747–754 10.1016/0076-6879(88)67086-8 3148842

[CR17] Frankel LK, Sallans L, Bellamy H et al (2013) Radiolytic mapping of solvent-contact surfaces in photosystem II of higher plants. J Biol Chem 288:23565–23572. 10.1074/jbc.M113.487033 23814046 10.1074/jbc.M113.487033 PMC3949330

[CR18] Gabdulkhakov A, Guskov A, Broser M et al (2009) Probing the accessibility of the Mn_4_Ca cluster in photosystem II: channels calculation, noble gas derivatization, and cocrystallization with DMSO. Structure 17:1223–1234. 10.1016/j.str.2009.07.010 19748343 10.1016/j.str.2009.07.010

[CR19] Gasanov R, Aliyeva S, Arao S et al (2007) Comparative study of the water oxidizing reactions and the millisecond delayed chlorophyll fluorescence in photosystem II at different pH. J Photochem Photobiol B 86:160–164. 10.1016/j.jphotobiol.2006.08.008 17067808 10.1016/j.jphotobiol.2006.08.008

[CR20] Geijer P, Morvaridi F, Styring S (2001) The S_3_ state of the oxygen-evolving complex in photosystem II is converted to the S_2_Y_Z_^•^ state at alkaline pH. Biochemistry 40:10881–10891. 10.1021/bi010040v 11535065 10.1021/bi010040v

[CR21] Gerwert K, Freier E, Wolf S (2014) The role of protein-bound water molecules in microbial rhodopsins. Biochim Biophys Acta BBA - Bioenerg 1837:606–613. 10.1016/j.bbabio.2013.09.006 10.1016/j.bbabio.2013.09.006 24055285

[CR22] Gisriel CJ, Wang J, Liu J et al (2022) High-resolution cryo-electron microscopy structure of photosystem II from the mesophilic cyanobacterium, synechocystis Sp. PCC 6803. Proc Natl Acad Sci 119:e2116765118. 10.1073/pnas.2116765118 34937700 10.1073/pnas.2116765118 PMC8740770

[CR23] Greife P, Schönborn M, Capone M et al (2023) The electron–proton bottleneck of photosynthetic oxygen evolution. Nature 1–6. 10.1038/s41586-023-06008-5 10.1038/s41586-023-06008-5 PMC10191853 37138082

[CR24] Guo Y, He L, Ding Y et al (2024) Closing Kok’s cycle of Nature’s water oxidation catalysis. Nat Commun 15:5982. 10.1038/s41467-024-50210-6 39013902 10.1038/s41467-024-50210-6 PMC11252165

[CR25] Haumann M, Müller C, Liebisch P et al (2005) Structural and oxidation state changes of the photosystem II manganese complex in four transitions of the water oxidation cycle (S_0_→S_1_, S_1_→S_2_, S_2_→S_3_, and S_3_,_4_→S_0_) characterized by X-ray absorption spectroscopy at 20 K and room temperature. Biochemistry 44:1894–1908. 10.1021/bi048697e 15697215 10.1021/bi048697e

[CR28] Hillier W, Wydrzynski T (2000) The affinities for the two substrate water binding sites in the O_2_ evolving complex of photosystem II vary independently during S-State turnover. Biochemistry 39:4399–4405. 10.1021/bi992318d 10757989 10.1021/bi992318d

[CR26] Hillier W, Wydrzynski T (2001) Oxygen ligand exchange at metal sites– implications for the O_2_ evolving mechanism of photosystem II. Biochim Biophys Acta BBA - Bioenerg 1503:197–209. 10.1016/S0005-2728(00)00225-5 10.1016/s0005-2728(00)00225-5 11115634

[CR27] Hillier W, Wydrzynski T (2008) ^18^O-Water exchange in photosystem II: substrate binding and intermediates of the water splitting cycle. Coord Chem Rev 252:306–317. 10.1016/j.ccr.2007.09.004

[CR29] Ho FM, Styring S (2008) Access channels and methanol binding site to the CaMn_4_ cluster in photosystem II based on solvent accessibility simulations, with implications for substrate water access. Biochim Biophys Acta BBA - Bioenerg 1777:140–153. 10.1016/j.bbabio.2007.08.009 10.1016/j.bbabio.2007.08.009 17964532

[CR30] Huang H-H, Camsund D, Lindblad P, Heidorn T (2010) Design and characterization of molecular tools for a synthetic biology approach towards developing cyanobacterial biotechnology. Nucleic Acids Res 38:2577–2593. 10.1093/nar/gkq164 20236988 10.1093/nar/gkq164 PMC2860132

[CR31] Hughes CS, Foehr S, Garfield DA et al (2014) Ultrasensitive proteome analysis using paramagnetic bead technology. Mol Syst Biol 10:757. 10.15252/msb.20145625 25358341 10.15252/msb.20145625 PMC4299378

[CR32] Hughes CS, Moggridge S, Müller T et al (2019) Single-pot, solid-phase-enhanced sample preparation for proteomics experiments. Nat Protoc 14:68–85. 10.1038/s41596-018-0082-x 30464214 10.1038/s41596-018-0082-x

[CR34] Hussein R, Ibrahim M, Bhowmick A et al (2021) Structural dynamics in the water and proton channels of photosystem II during the S_2_ to S_3_ transition. Nat Commun 12:6531. 10.1038/s41467-021-26781-z 34764256 10.1038/s41467-021-26781-z PMC8585918

[CR35] Hussein R, Ibrahim M, Bhowmick A et al (2023) Evolutionary diversity of proton and water channels on the oxidizing side of photosystem II and their relevance to function. Photosynth Res 158:91–107. 10.1007/s11120-023-01018-w 37266800 10.1007/s11120-023-01018-w PMC10684718

[CR33] Hussein R, Graça A, Forsman J et al (2024) Cryo–electron microscopy reveals hydrogen positions and water networks in photosystem II. Science 384:1349–1355. 10.1126/science.adn6541 38900892 10.1126/science.adn6541

[CR36] Ibrahim M, Fransson T, Chatterjee R et al (2020) Untangling the sequence of events during the S_2_→S_3_ transition in photosystem II and implications for the water oxidation mechanism. Proc Natl Acad Sci U S A 117:12624–12635. 10.1073/pnas.2000529117 32434915 10.1073/pnas.2000529117 PMC7293653

[CR37] Isobe H, Shoji M, Yamanaka S et al (2012) Theoretical illumination of water-inserted structures of the CaMn_4_O_5_ cluster in the S_2_ and S_3_ States of oxygen-evolving complex of photosystem II: full geometry optimizations by B3LYP hybrid density functional. Dalton Trans 41:13727–13740. 10.1039/C2DT31420G 23037319 10.1039/c2dt31420g

[CR38] Isobe H, Suzuki T, Suga M et al (2024) Conformational flexibility of D1-Glu189: A crucial determinant in substrate water selection, positioning, and stabilization within the oxygen-evolving complex of photosystem II. ACS Omega. 10.1021/acsomega.4c09981 39713658 10.1021/acsomega.4c09981 PMC11656237

[CR39] Kern J, Chatterjee R, Young ID et al (2018) Structures of the intermediates of Kok’s photosynthetic water oxidation clock. Nature 563:421–425. 10.1038/s41586-018-0681-2 30405241 10.1038/s41586-018-0681-2 PMC6485242

[CR40] Kim CJ, Debus RJ (2019) One of the substrate waters for O_2_ formation in photosystem II is provided by the water-splitting Mn_4_CaO_5_ cluster’s Ca^2^^ + ^Ion. Biochemistry 58:3185–3192. 10.1021/acs.biochem.9b00418 31276397 10.1021/acs.biochem.9b00418

[CR41] Kim CJ, Debus RJ (2020) Roles of D1-Glu189 and D1-Glu329 in O_2_ formation by the water-splitting Mn_4_Ca cluster in photosystem II. Biochemistry 59:3902–3917. 10.1021/acs.biochem.0c00541 32931699 10.1021/acs.biochem.0c00541

[CR42] Krewald V, Retegan M, Cox N et al (2015) Metal oxidation States in biological water splitting. Chem Sci 6:1676–1695. 10.1039/C4SC03720K 29308133 10.1039/c4sc03720k PMC5639794

[CR43] Kulik LV, Epel B, Lubitz W, Messinger J (2007) Electronic structure of the Mn_4_O_x_Ca cluster in the S_0_ and S_2_ States of the oxygen-evolving complex of photosystem II based on pulse ^55^Mn-ENDOR and EPR spectroscopy. J Am Chem Soc 129:13421–13435. 10.1021/ja071487f 17927172 10.1021/ja071487f

[CR44] Li H, Nakajima Y, Nango E et al (2024) Oxygen-evolving photosystem II structures during S_1_–S_2_–S_3_ transitions. Nature 626:670–677. 10.1038/s41586-023-06987-5 38297122 10.1038/s41586-023-06987-5 PMC10866707

[CR45] Liang F, Englund E, Lindberg P, Lindblad P (2018) Engineered cyanobacteria with enhanced growth show increased ethanol production and higher biofuel to biomass ratio. Metab Eng 46:51–59. 10.1016/j.ymben.2018.02.006 29477858 10.1016/j.ymben.2018.02.006

[CR46] Liu K, Brown MG, Cruzan JD, Saykally RJ (1996) Vibration-rotation tunneling spectra of the water pentamer: structure and dynamics. Science 271:62–64. 10.1126/science.271.5245.62

[CR47] Lundberg M, Blomberg MRA, Siegbahn PEM (2003) Modeling water exchange on monomeric and dimeric Mn centers. Theor Chem Acc 110:130–143. 10.1007/s00214-003-0474-y

[CR48] Mamedov F, Sayre RT, Styring S (1998) Involvement of histidine 190 on the D1 protein in electron/proton transfer reactions on the donor side of photosystem II. Biochemistry 37:14245–14256. 10.1021/bi980194j 9760263 10.1021/bi980194j

[CR49] Mamedov F, Stefansson H, Albertsson P-Å, Styring S (2000) Photosystem II in different parts of the thylakoid membrane: A functional comparison between different domains. Biochemistry 39:10478–10486. 10.1021/bi992877k 10956038 10.1021/bi992877k

[CR50] Maurer M, Oostenbrink C (2019) Water in protein hydration and ligand recognition. J Mol Recognit 32:e2810. 10.1002/jmr.2810 31456282 10.1002/jmr.2810 PMC6899928

[CR51] Messinger J (2004) Evaluation of different mechanistic proposals for water oxidation in photosynthesis on the basis of Mn4OxCa structures for the catalytic site and spectroscopic data. Phys Chem Chem Phys 6:4764–4771. 10.1039/B406437B

[CR52] Messinger J, Badger M, Wydrzynski T (1995) Detection of one slowly exchanging substrate water molecule in the S_3_ state of photosystem II. Proc Natl Acad Sci 92:3209–3213. 10.1073/pnas.92.8.3209 11607525 10.1073/pnas.92.8.3209 PMC42135

[CR53] Messinger J, Nugent JHA, Evans MCW (1997a) Detection of an EPR multiline signal for the S_0_* state in photosystem II. Biochemistry 36:11055–11060. 10.1021/bi9711285 9333322 10.1021/bi9711285

[CR54] Messinger J, Robblee JH, Yu WO et al (1997b) The S_0_ state of the oxygen-evolving complex in photosystem II is paramagnetic: detection of an EPR multiline signal. J Am Chem Soc 119:11349–11350. 10.1021/JA972696A 25221336 10.1021/ja972696a PMC4161286

[CR55] Nagao R, Ueoka-Nakanishi H, Noguchi T (2017) D1-Asn-298 in photosystem II is involved in a hydrogen-bond network near the redox-active tyrosine YZ for proton exit during water oxidation. J Biol Chem 292:20046–20057. 10.1074/jbc.M117.815183 29046348 10.1074/jbc.M117.815183 PMC5723994

[CR56] Navarro MP, Ames WM, Nilsson H et al (2013) Ammonia binding to the oxygen-evolving complex of photosystem II identifies the solvent-exchangeable oxygen bridge (µ-oxo) of the manganese tetramer. Proc Natl Acad Sci U S A 110:15561–15566. 10.1073/PNAS.1304334110/SUPPL_FILE/PNAS.201304334SI.PDF 24023065 10.1073/pnas.1304334110 PMC3785721

[CR57] Nixon PJ, Diner BA (1992) Aspartate ^17^o of the photosystem II reaction center polypeptide D1 is involved in the assembly of the oxygen-evolving manganese cluster. Biochemistry 31:942–948. 10.1021/bi00118a041 1731951 10.1021/bi00118a041

[CR58] Noguchi T (2007) FTIR detection of water reactions in the oxygen-evolving centre of photosystem II. Philos Trans R Soc B Biol Sci 363:1189–1195. 10.1098/rstb.2007.2214 10.1098/rstb.2007.2214 PMC2614091 17965007

[CR59] Okamoto Y, Shimada Y, Nagao R, Noguchi T (2021) Proton and water transfer pathways in the S_2_→S_3_ transition of the water-Oxidizing complex in photosystem II: time-resolved infrared analysis of the effects of D1-N298A mutation and NO_3_^–^ substitution. J Phys Chem B 125:6864–6873. 10.1021/acs.jpcb.1c03386 34152151 10.1021/acs.jpcb.1c03386

[CR60] Pantazis DA, Ames W, Cox N et al (2012) Two interconvertible structures that explain the spectroscopic properties of the oxygen-evolving complex of photosystem II in the S_2_ state. Angew Chem - Int Ed 51:9935–9940. 10.1002/anie.201204705 10.1002/anie.201204705 22907906

[CR61] Pushkar Y, Ravari K, Jensen A, Palenik SC M (2019) Early binding of substrate oxygen is responsible for a spectroscopically distinct S_2_ state in photosystem II. J Phys Chem Lett 10:5284–5291. 10.1021/acs.jpclett.9b01255 31419136 10.1021/acs.jpclett.9b01255

[CR62] Rapatskiy L, Cox N, Savitsky A et al (2012) Detection of the water-binding sites of the oxygen-evolving complex of photosystem ii using W-Band ^17^O electron–electron double resonance-detected NMR spectroscopy. J Am Chem Soc 134:16619–16634. 10.1021/ja3053267 22937979 10.1021/ja3053267

[CR63] Rappaport F, Diner BA (2008) Primary photochemistry and energetics leading to the oxidation of the (Mn)4Ca cluster and to the evolution of molecular oxygen in photosystem II. Coord Chem Rev 252:259–272. 10.1016/j.ccr.2007.07.016

[CR64] Service RJ, Hillier W, Debus RJ (2010) Evidence from FTIR difference spectroscopy of an extensive network of hydrogen bonds near the Oxygen-Evolving Mn_4_Ca cluster of photosystem II involving D1-Glu65, D2-Glu312, and D1-Glu329. Biochemistry 49:6655–6669. 10.1021/bi100730d 20593803 10.1021/bi100730d PMC2917469

[CR65] Service RJ, Yano J, McConnell I et al (2011) Participation of glutamate-354 of the CP43 polypeptide in the ligation of manganese and the binding of substrate water in photosystem II. Biochemistry 50:63–81. 10.1021/bi1015937 21114287 10.1021/bi1015937 PMC3089812

[CR66] Shen J-R (2015) The structure of photosystem II and the mechanism of water oxidation in photosynthesis. Annu Rev Plant Biol 66:23–48. 10.1146/annurev-arplant-050312-120129 25746448 10.1146/annurev-arplant-050312-120129

[CR67] Shevela D, Kern JF, Govindjee G, Messinger J (2023) Solar energy conversion by photosystem II: principles and structures. Photosynth Res 156:279–307. 10.1007/s11120-022-00991-y 36826741 10.1007/s11120-022-00991-y PMC10203033

[CR68] Shoji M, Isobe H, Yamaguchi K (2015) QM/MM study of the S_2_ to S_3_ transition reaction in the oxygen-evolving complex of photosystem II. Chem Phys Lett 636:172–179. 10.1016/j.cplett.2015.07.039 10.1021/acs.jpcb.5b05740 26440915

[CR69] Siegbahn PEM (2006) O-O bond formation in the S_4_ state of the oxygen-evolving complex in photosystem II. Chem– Eur J 12:9217–9227. 10.1002/chem.200600774 17029313 10.1002/chem.200600774

[CR70] Siegbahn PEM (2009) Structures and energetics for O_2_ formation in photosystem II. Acc Chem Res 42:1871–1880. 10.1021/ar900117k 19856959 10.1021/ar900117k

[CR71] Siegbahn PEM (2013) Substrate water exchange for the oxygen evolving complex in PSII in the S_1_, S_2_, and S_3_ States. J Am Chem Soc 135:9442–9449 23742698 10.1021/ja401517e

[CR72] Suga M, Akita F, Sugahara M et al (2017) Light-induced structural changes and the site of O = O bond formation in PSII caught by XFEL. Nature 543:131–135. 10.1038/nature21400 28219079 10.1038/nature21400

[CR73] Suga M, Akita F, Yamashita K et al (2019) An oxyl/oxo mechanism for oxygen-oxygen coupling in PSII revealed by an x-ray free-electron laser. Science 366:334–338. 10.1126/SCIENCE.AAX6998/SUPPL_FILE/AAX6998_SUGA_SM.PDF 31624207 10.1126/science.aax6998

[CR74] Sugiura M, Rappaport F, Hillier W et al (2009) Evidence that D1-His332 in photosystem II from thermosynechococcus elongatus interacts with the S_3_-state and not with the S_2_-state. Biochemistry 48:7856–7866. 10.1021/bi901067b 19624137 10.1021/bi901067b

[CR75] Tso J, Sivaraja M, Dismukes GC (1991) Calcium limits substrate accessibility or reactivity at the manganese cluster in photosynthetic water oxidation. Biochemistry 30:4734–4739. 10.1021/bi00233a014 1851435 10.1021/bi00233a014

[CR76] Umena Y, Kawakami K, Shen J-R, Kamiya N (2011) Crystal structure of oxygen-evolving photosystem II at a resolution of 1.9 Å. Nature 473:55–60. 10.1038/nature09913 21499260 10.1038/nature09913

[CR77] Vass I, Kirilovsky D, Etienne A-L (1999) UV-B radiation-induced donor- and acceptor-side modifications of photosystem ii in the cyanobacterium synechocystis sp. PCC 6803. Biochemistry 38:12786–12794. 10.1021/bi991094w 10504248 10.1021/bi991094w

[CR78] Vassiliev S, Comte P, Mahboob A, Bruce D (2010) Tracking the flow of water through photosystem II using molecular dynamics and streamline tracing. Biochemistry 49:1873–1881. 10.1021/bi901900s 20121111 10.1021/bi901900s

[CR79] Vassiliev S, Zaraiskaya T, Bruce D (2012) Exploring the energetics of water permeation in photosystem II by multiple steered molecular dynamics simulations. Biochim Biophys Acta BBA - Bioenerg 1817:1671–1678. 10.1016/j.bbabio.2012.05.016 10.1016/j.bbabio.2012.05.016 22683291

[CR81] Volgusheva A, Styring S, Mamedov F (2013) Increased photosystem II stability promotes H_2_ production in sulfur-deprived chlamydomonas reinhardtii. Proc Natl Acad Sci 110:7223–7228. 10.1073/pnas.1220645110 23589846 10.1073/pnas.1220645110 PMC3645517

[CR80] Volgusheva A, Kruse O, Styring S, Mamedov F (2016) Changes in the photosystem II complex associated with hydrogen formation in sulfur deprived *Chlamydomonas reinhardtii*. Algal Res 18:296–304. 10.1016/j.algal.2016.06.025

[CR82] Weisz DA, Gross ML, Pakrasi HB (2017) Reactive oxygen species leave a damage trail that reveals water channels in photosystem II. Sci Adv 3:eaao3013. 10.1126/sciadv.aao3013 29159285 10.1126/sciadv.aao3013 PMC5693562

[CR83] Williams JGK (1988) Construction of specific mutations in photosystem II photosynthetic reaction center by genetic engineering methods in Synechocystis 6803. In: Methods in Enzymology. Academic Press, pp 766–778

[CR84] Wydrzynski T, Hillier W, Messinger J (1996) On the functional significance of substrate accessibility in the photosynthetic water oxidation mechanism. Physiol Plant 96:342–350. 10.1111/j.1399-3054.1996.tb00224.x

[CR85] Xu T, Bin X, Kirk SR et al (2020) Flip rearrangement in the water pentamer: analysis of electronic structure. Int J Quantum Chem 120:e26124. 10.1002/qua.26124

