# Supplementary material for: Probing substrate water access through the O1 channel of Photosystem II by single site mutations and membrane inlet mass spectrometry
Source: Photosynth Res. 2025 Apr 22;163(3):28. doi: 10.1007/s11120-025-01147-4 (PMC12014804; doi:10.1007/s11120-025-01147-4)
Supplement: Supplementary file 1 — Supplementary Material 1 [file 11120_2025_1147_MOESM1_ESM.pdf]

Supporting information for:

Probing substrate water access through the O1 channel of Photosystem II by single site mutations and membrane inlet mass spectrometry

A. Orkun Aydin[1], Casper de Lichtenberg[1], Feiyan Liang[1,2], Jack Forsman[3,4], André T. Graça[3,5], Petko Chernev[1], Shaochun Zhu[3], André Mateus[3,6], Ann Magnuson[1], Mun Hon Cheah[1], Wolfgang P. Schröder[3,4], Felix Ho[1], Peter Lindblad[1], Richard J. Debus[7], Fikret Mamedov[1] and Johannes Messinger[1,4*]

Addresses

[1] Molecular Biomimetics, Department of Chemistry – Ångström, Uppsala University, 751 20 Uppsala, Sweden

[2] present address: Department of Plant and Environmental Sciences, University of Copenhagen, 1871 Frederiksberg C, Denmark

[3] Department of Chemistry, Chemical Biology Centre, Umeå University, 907 36 Umeå, Sweden

[4] Umeå Plant Science Center (UPSC), Department of Plant Physiology, Umeå University, 901 87 Umeå, Sweden

[5] present address: European Molecular Biology Laboratory, EMBL Grenoble, 38042 Grenoble, France

[6] The Laboratory for Molecular Infection Medicine Sweden (MIMS), Umeå University, 907 36 Umeå, Sweden

[7] Department of Biochemistry, University of California, Riverside, CA 92521, USA

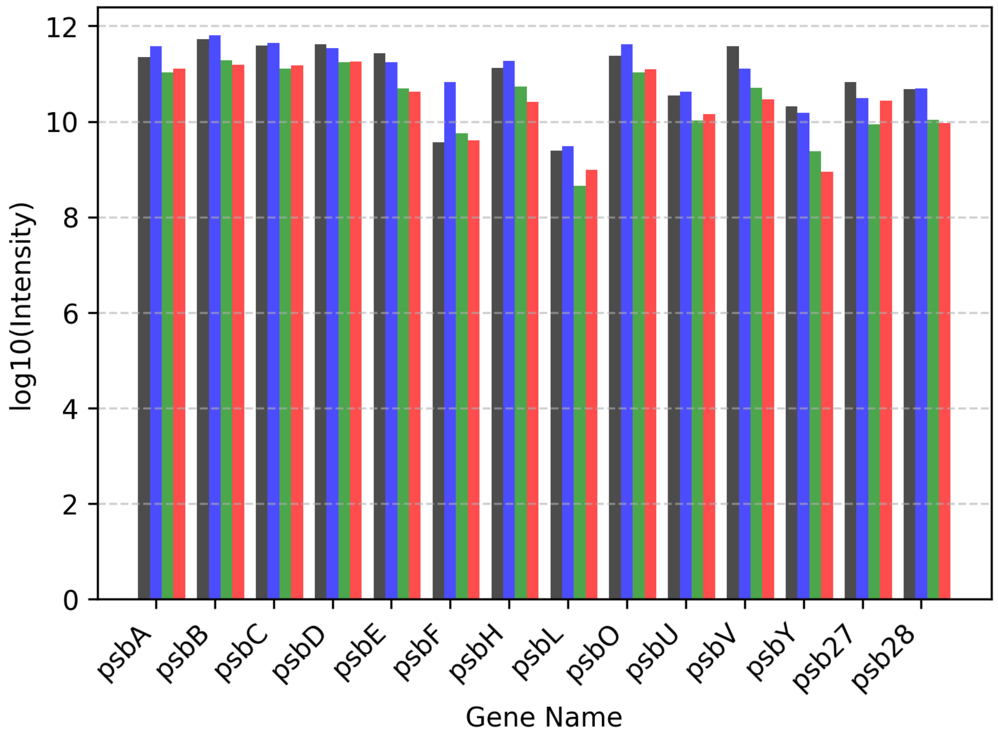

Figure S1 Protein subunit composition of isolated PSII core complexes from wild-type (WT) and the E329F, E329L, and V410S mutants of *Synechocystis*. The log10 of the summed intensities of the three most abundant peptides per protein, which were obtained by trypsin digestion, are depicted for subunits of WT *(black)*, E329F *(blue)*, E329L *(green)*, and V410S *(red)*. The reaction center proteins psbA (D1), psbB (CP47), psbC (CP43), and psbD (D2) are indicated, along with the lumenal extrinsic subunits psbO, psbU, and psbV. Species comparison is best done for each protein since the log10(intensity) is only approximately comparable between different proteins as it depends on the physical and chemical properties of the peptides. For experimental details, see Material and Methods. The full mass spectrometry proteomics data have been deposited to the ProteomeXchange Consortium via the PRIDE (Perez Riverol et al. 2025) partner repository with the dataset identifier PXD061921.

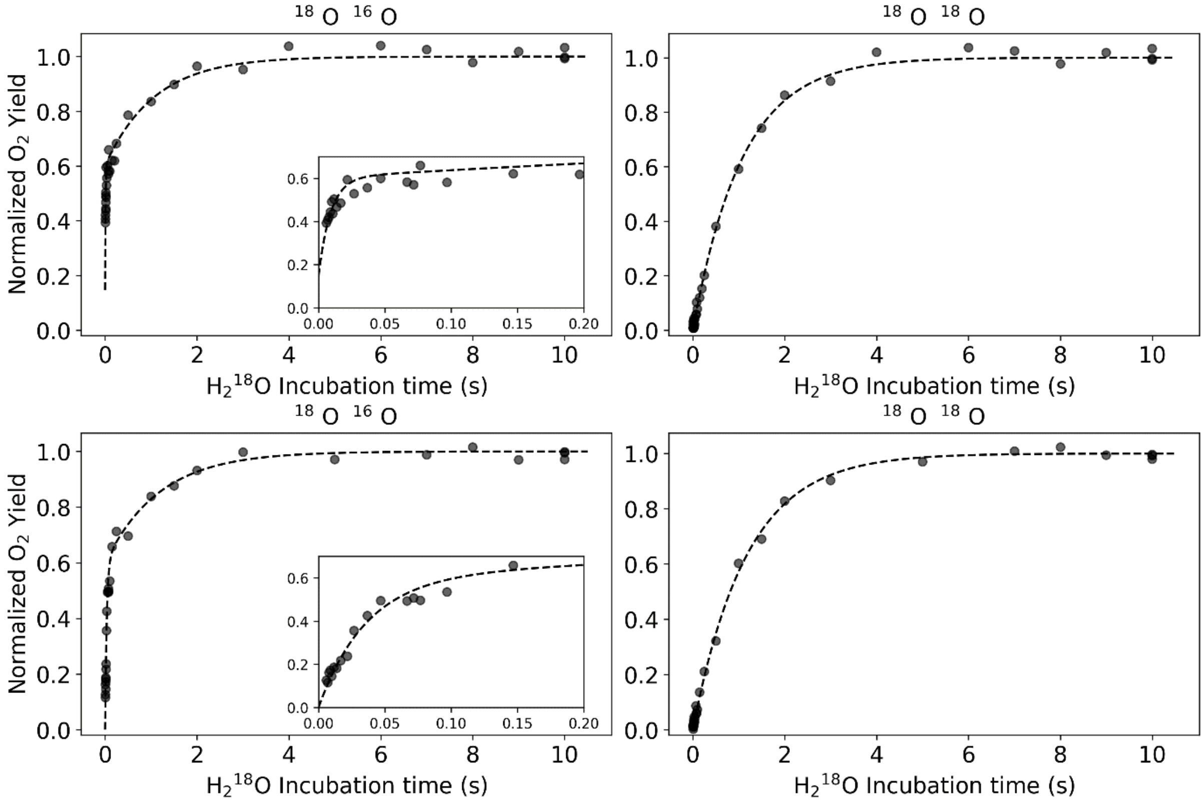

Figure S2 Substrate water exchange kinetics of wild type (WT) PSII core complexes isolated from *Synechcystis*. Symbols represent independent data points, while the fits are represented by dashed lines (for parameters, see Table 1). The insets in m/z 34 graphs shows an expansion of the fast phase of substrate water exchange reflecting W_f exchange. The data were recorded at 10°C and pH 6.5.

Table S1 Comparison of substrate exchange rates obtained by applying the corrections for the rate of mixing of the labeled water with the sample in the TR-MIMS cell and the isotope exchange exchange occurring in the S_3 state, which contributes to the measured S_2 state exchange kinetics, as we described earlier (Chernev et al. 2024). The rates are in line with previously reported values for the cases without the non-instant mixing correction for additional exchange occurring S_3 state during the time interval between second and third flashes (de Lichtenberg et al. 2021). Data were obtained at at 10 °C and pH 6.5.

Study	Non-instant Mixing	S_3-Exchange	S_3		S_2		chi-squared
			k_f	k_s	k_f	k_s	
1	-	-	23.4	0.76			-
2	-	-	24.0	0.85			0.028
2	+	-	28.0	0.85			0.033
1	-	+			84	0.97	-
2	-	+			84	0.92	0.044
2	-	-			104	0.92	0.047
2	+	-			145	0.92	0.055
2	+	+			111	0.93	0.048

[1] (de Lichtenberg et al., 2021).
[2] This study.

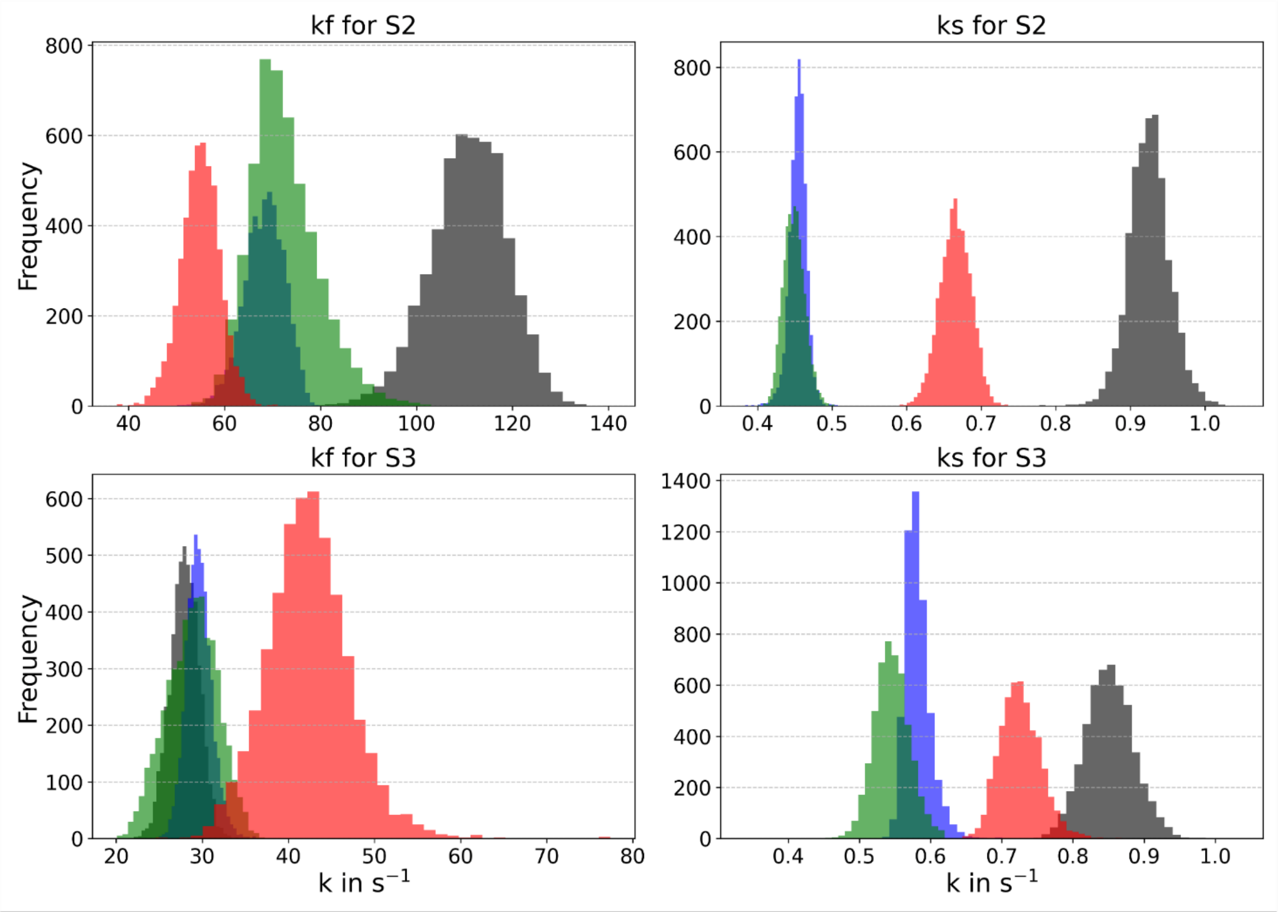

Figure S3 The results of variability analysis using the jackknife resampling method (Efron and Tibshirani 1994). The procedure involves resampling the dataset by removing a datapoint and duplicating another one randomly to before performing the parameter optimization, to synthetically add weight on a datapoint while removing on another. This procedure is repeated 5000 times for each dataset to get the variability in the k_f and k_s parameters. In each histogram, y-axis corresponds to the number of occurrences, namely the frequency, of the corresponding k-value in the x-axis obtained during individual fitting procedure. WT, E329F, E329L and V410S data are shown in black, blue, green and red, respectively. The error margin for each optimized parameter can then be estimated as one standard deviation distanced value from the center.

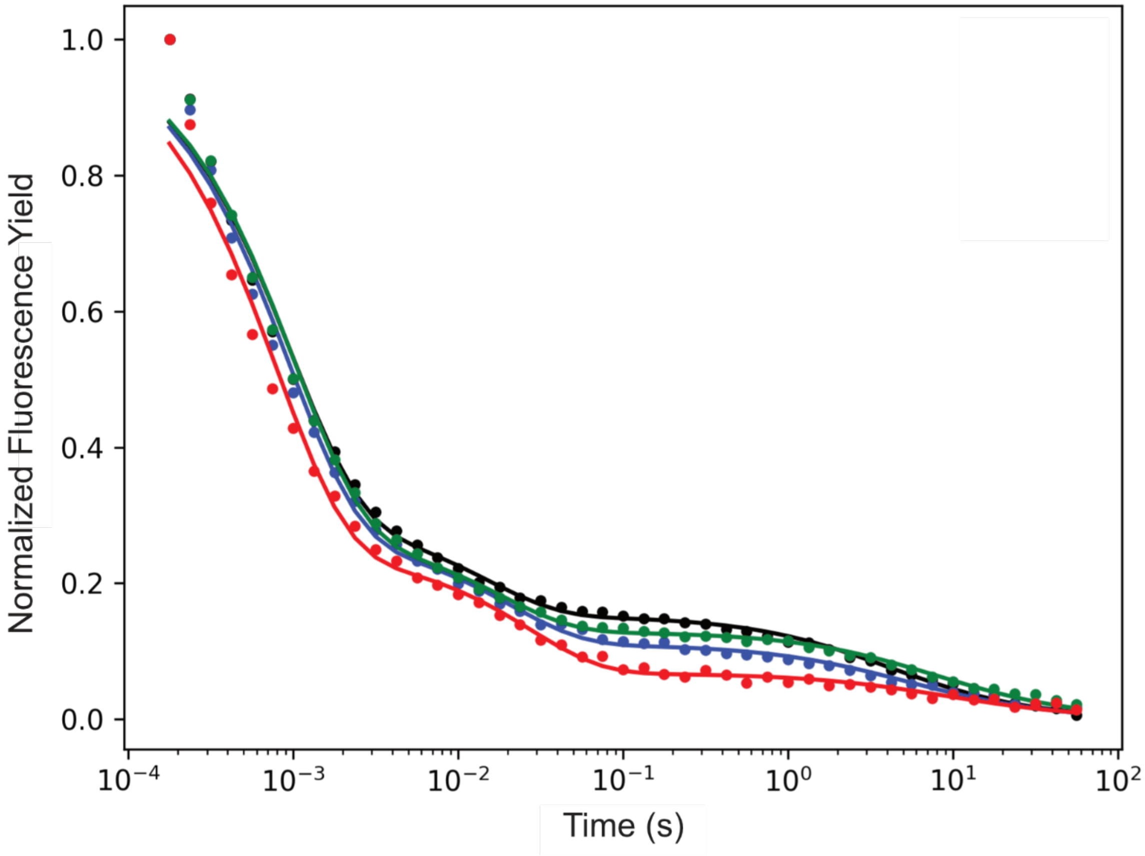

Figure S4 Normalized variable fluorescence decay kinetics recorded after a single actinic flash applied on whole cells. The lines denote the three exponential fit model optimized for the datasets shown with spheres. WT, E329F, E329L and V410S are shown with colors; black, blue, green and red, respectively. Normalized variable Fluorescence decay kinetics record after a single actinic flash applied to the WT, D1-E329F, D1-E329L and CP43-V410S whole cell samples without the addition of DCMU.

Table S2 Fits of the variable fluorescence data of WT, E329F, E329L and V410S displayed in Figure S4 that were obtained in absence of DCMU.

Samples	Fast Phase	Middle Phase	Slow Phase
	τ_1 (ms) [amp (%)]	τ_2 (ms) [amp (%)]	τ_3 (s) [amp (%)]
WT	953 ± 69 (71%)	17 ± 7 (15%)	4.9 ± 1.7 (13%)
D1-E329F	924 ± 60 (73%)	21 ± 7 (16%)	4.2 ± 2.2 (8%)
D1-E329L	995 ± 65 (73%)	18 ± 7 (14%)	6.3 ± 2.7 (10%)
CP43-V410S	791 ± 53 76%)	26 ± 9 (18%)	4.9 ± 5.3 (5%)

SI References

Chernev P, Aydin AO, Messinger J (2024) On the simulation and interpretation of substrate-water exchange experiments in photosynthetic water oxidation. Photosynth Res. https://doi.org/10.1007/s11120-024-01084-8

de Lichtenberg C, Kim CJ, Chernev P, et al (2021) The exchange of the fast substrate water in the S 2 state of photosystem II is limited by diffusion of bulk water through channels – implications for the water oxidation mechanism. Chem Sci 12:12763–12775. https://doi.org/10.1039/D1SC02265B

Efron B, Tibshirani RJ (1994) An Introduction to the Bootstrap. Chapman and Hall/CRC, New York

Perez-Riverol Y, Bandla C, Kundu DJ, Kamatchinathan S, Bai J, Hewapathirana S, John NS, Prakash A, Walzer M, Wang S, Vizcaíno JA. The PRIDE database at 20 years: 2025 update. Nucleic Acids Res. 2025 Jan 6;53(D1):D543-D553. doi: 10.1093/nar/gkae1011.
